# A systematic literature review and meta‐analysis of virtual reality nature effects on higher education students' mental health and wellbeing

**DOI:** 10.1111/aphw.70060

**Published:** 2025-09-04

**Authors:** Gill Hubbard, Philip Albert Verde, Alexia Barrable, Chris O'Malley, Nicholas Barnes, Paul Toner

**Affiliations:** ^1^ School of Health Sciences, 11 Airlie Place University of Dundee Dundee Scotland UK; ^2^ NHS Education for Scotland Edinburgh Scotland UK; ^3^ Division of Psychology, Sociology and Education Queen Margaret University Edinburgh Scotland UK; ^4^ University of the Highlands and Islands Inverness Scotland UK; ^5^ Child and Adolescent Psychiatrist NHS Highlands, Assynt House, Beechwood Park Inverness Scotland UK

**Keywords:** anxiety, cognition, depression, health psychology, higher education, interventions, mental health, mood, nature, stress, students, virtual reality, well‐being

## Abstract

Virtual Reality nature (VRn) may deliver mental health and wellbeing without being outside in real nature. The main objective of this systematic review and meta‐analysis was to report effects of VRn on mental health and wellbeing of students in higher education. To be eligible, participants were higher education students, the intervention was VRn, the outcome variable was a mental health parameter, and the study design was experimental. Information sources were: OVID (Embase, MEDLINE, PsycINFO), SCOPUS, CINAHL, Cochrane Library, PubMed and GreenFILE. Searches were conducted May 2023 and re‐run November 2024. The modified Downs and Black checklist for randomised and non‐randomised studies was used to assess risk of bias. To synthesise results, data were first extracted into a Microsoft Excel spreadsheet and summarised in narrative, statistical and tabular formats. Twenty‐four articles were included. Two studies were ‘good’ quality, 18 ‘fair’, and four ‘poor’. Total sample size was 1,419. Two studies compared VRn versus real nature, six VRn versus no intervention, seven VRn versus flat‐screen. Twelve studies included forests as the only natural environment. Thirteen of 17, seven of eight, four of five, and four of seven studies reported pre‐post intervention beneficial effects on self‐reported mood, anxiety, stress and cognition, respectively. Nine of 13, six of 10 and all five studies that measured cardiovascular, skin conductivity and brain activity respectively, reported pre‐post intervention beneficial effects. All five studies that measured anxiety, all three studies that measured mood, and all four studies that measured stress, found no significant differences between VRn and flat‐screen images of nature. Caution is required drawing conclusions due to studies' quality and sample sizes. That said, the review suggests that nature replicated in VR shows promise for benefits to mental health and wellbeing in higher education students.

## INTRODUCTION

Good Health and Wellbeing is a United Nation’ Social Development Goal (SDG). In the context of the ‘One Health’ approach that aims to ‘sustainably balance and optimise the health of people, animals and ecosystems,’ (World Health Organisation, 2025) interventions that potentially enhance people's relationship and connection with nature may be valuable. Virtual reality nature (VRn) for instance, might offer benefits in contexts where real nature contact is limited or unavailable by supporting psychological aspects of human‐nature connectedness. VR is at the forefront of digital advancements in addressing mental health and wellbeing (Bucci et al., 2019; Cieślik et al., 2020; Di Pompeo et al., 2023; Kelson et al., 2021; Valmaggia et al., 2016; Wiebe et al., 2022). Virtual reality nature (VRn) may offer a route to *real* nature benefits for people who do not access *real* nature. There are for example, inequalities to accessing green space; in the UK people aged over 64 have the greatest access to green space with 31% living in neighbourhoods with the most access to green space, compared with 18% of 25 to 34‐year‐olds (The Health Foundation, 2024).

VRn creates the illusion of being transported into a world that appears to be real. Computer‐generated three‐dimensional environments (‘computer‐generated imagery’ [CGI]) are a synthetic still or animated physical environment, consisting of an interpretation and rendition of what natural objects, colours, shapes, motion and light effects look like, whereas real‐scene‐based 360^°^ videos are a replication of real nature, seen through the perspective of the individual who shot the film (White et al., 2018). Both CGI and 360^°^ videos can capture everything around a person in a full circle so they can look around in all directions. ‘Presence’ and ‘immersion’ in VRn are considered to be greater compared to static simulations (e.g., 2 Dimensional [2D] photographs) and are thus expected to render more beneficial health and wellbeing effects (de Kort et al., 2006). ‘Immersion’ has been defined as the objective level of sensory engagement by the VR resulting in a flow state, whereas ‘presence’ is the subjective psychological response of the user experiencing VR, resulting in a sense of ‘being’ (Berkman & Akan, 2019; Wilkinson et al., 2021). Other attributes of VR that may influence its impact on mental health and wellbeing include sensory components, such as auditory stimuli (soundscape). Visual and auditory stimuli are the two primary information sources people perceive from their surroundings (Preis et al., 2015) and greater congruence between these stimuli in VR has been linked to a person's sense of immersion and presence (Kim & Lee, 2022). However, there is no consensus on definition or measurement of these technical features of VR which limits our ability to understand relationships between VRn's ‘active ingredients’, which are the observable, reproducible and simplified components of an intervention designed to affect mental health and wellbeing.

### Psychological pathways linking VRn and mental health and wellbeing

Being exposed to *real* nature is known to elicit a range of benefits to human health (Bratman et al., 2019; Gascon et al., 2015; Meredith et al., 2019; White et al., 2021). Research about mental health and wellbeing benefits of being exposed to *real* nature is dominated by three main biopsychosocial theories: ‘biophilia hypothesis’ (Kellert & Wilson, 1993; Wilson, 1984), ‘stress recovery theory’ (Ulrich, 1983) and ‘attention restoration theory’ (Kaplan, 1995; Kaplan & Kaplan, 1989). These theories suggest that pathways to mental health and wellbeing are linked to nature's psychological and physiological relaxation functions alongside its inherent restorative qualities that shift people's emotion and cognition as a consequence of evolutionary processes (Beery et al., 2015). The biophilia hypothesis proposes that people have an innate tendency to affiliate with the natural biological world as a result of human evolution, which means that *real* nature de facto fulfils important psychological needs and improves mental wellbeing. The stress recovery theory proposes that nature reduces stress by eliciting positive emotional states and lowering physiological arousal and that these outcomes are a product of our evolutionary past that has resulted in predisposed responses to cues (e.g., vegetation) that once signalled better survival. The attention restoration theory proposes that natural environments restore the depletion of directed attention, which is a cognitive resource that is often depleted in modern urban life due to overstimulation and multisensory stimuli. Restorativeness is therefore a significant outcome for investigations of VRn. In support of these biopsychosocial theories and proposed pathways for mental health and wellbeing, studies summarised in systematic reviews have consistently shown that exposure to *real* nature have beneficial impacts on emotions and stress across a diverse range of natural settings (Berto, 2014; McMahan & Estes, 2015; Mygind et al., 2019). Psychological and physiological relaxation improvements have been detected via objective physiological indices (e.g., heart rate variability, electrodermal activity and saliva cortisol) and self‐reported questionnaires (e.g., the Positive and Negative Affect Schedule and the State‐ Trait Inventory) (H. Li, Dong, et al., 2021). The parasympathetic branch of the autonomic nervous system regulates relaxation and heart rate variability reflects parasympathetic activation. Studies have also evaluated restorativeness, which indexes restoration from attention fatigue (Moll et al., 2022).

VRn can deliver these mental health and wellbeing benefits via immersive nature experience and represents a potentially inexpensive and convenient way to provide access to the benefits of *real* nature without being physically outside in nature. Recent reviews have reported virtual nature's (VR and 2D) beneficial effects on mental health and wellbeing (Browning et al., 2021, Browning, Shipley, et al., 2020, Frost et al., 2022, Li, Ding, et al., 2022). Browning and colleagues examined 175 experiments within 148 research papers, with mainly student samples and found that 100 experiments demonstrated mood improvements and 50 experiments were associated with improved cognition or attention, perceived restoration, reductions in stress and increased pain tolerance (Browning et al., 2021). The related meta‐analysis found that six studies revealed how real nature improved mood more than simulated nature, whereas, both real and simulated nature reduced negative affect (Browning, Shipley, et al., 2020). However, the review and meta‐analysis by Browning and colleagues were not just limited to VR but included 2D photographs, slideshows and videos, which limits our understanding of VRn's impact on mental health and wellbeing specifically. A systematic review of 21 peer‐reviewed research studies, with one‐third of all studies comprised predominantly of student populations, found that virtual nature decreased negative affect but did not improve positive affect, although some studies found no change or even an increase in negative affect (Frost et al., 2022). A systematic review and meta‐analysis of 28 studies from 26 publications, 16 of which were conducted with a mix of students and university or institution employees, found an association between immersion in a simulated natural environment and increased positive affect and that the level of immersion explained the heterogeneity of positive affect (Li, Ding, et al., 2022). However, these two latter reviews highlight significant research gaps, such as, limited attention paid to the technical features of VRn and their mechanisms of action to elucidate the association between virtual nature and mental health (Li, Ding, et al., 2022). Our understanding and ability to replicate health benefits gained from *real* nature in virtual nature and more specifically in VR, is therefore limited at present, and further primary research and systematic reviews about VRn are thus warranted.

### Mental health in higher education students

Restricting a systematic review to a specific population can offer several advantages, including increased relevance, enhanced precision due to minimising heterogeneity and a more focused analysis, ultimately leading to more actionable conclusions. A major disadvantage of course, is that this restriction to a specific population reduces generalisability of the findings to other populations. This systematic review is restricted to studies involving students in higher education. There are several reasons for restricting the review to this specific population. First, some of the main causes of poor mental health vary by age (Gondek et al., 2024) and poor mental health in the student population is related to context. A 2023 UK survey found that young people cited different reasons for feeling anxious but issues around college or university were the most common – mentioned by two in five (40%) of young people (Mental Health Foundation, 2023). A 2017 survey of over 19,500 university students in the UK found that 33% of students had a previous personal, emotional, behavioural or mental health problem that they felt needed professional help, and 69% of students had suffered with worry or anxiety (Pereira et al., 2018). The Covid‐19 pandemic further exacerbated mental health problems in the student population; systematic reviews and meta‐analyses showed rates of anxiety and depression between 30 and 41% (Batra et al., 2021, Chang et al., 2021, Y. Li, Wang, et al., 2021, Liyanage et al., 2021). A 2022 UK survey concluded that the disruption caused by Covid‐19 may keep student anxiety levels raised for some time yet (Neves & Brown, 2022). Poor mental health has a detrimental impact on academic learning which will then likely have longer term implications for life success (Pascoe et al., 2020). Therapeutic interventions to reduce symptoms of anxiety, stress and low mood in higher education students are therefore required both to improve students' mental health and wellbeing as well as to support their learning and academic outcomes.

Reviewing recent evidence of the effects of VRn on student mental health and wellbeing will inform decisions about its potential future usefulness and implementation in higher education settings. VRn could, for instance, be used as a quick stress‐recovery method during lesson breaks or between exams if Head‐Mounted Devices (HMDs) are available in cafes and libraries alongside encouraging students to go outdoors if green space is readily accessible.

### Objectives

The main objective of this systematic narrative review of literature was to address the following question: What are the effects of VRn on mental health and wellbeing of students in higher education? Further objectives were to describe theories and reasons used by authors to justify their study of the relationship between VRn and human health, VRn intervention components (‘active ingredients’) and the results of investigations of relationships between VR technical (e.g., interactive activities vs. none) and different kinds of natural environments (e.g. forest, beach) components and outcomes so that we can improve understandings of the underlying mechanisms by which VRn influences mental health and wellbeing. The PRISMA 2020 checklist was used to structure this article (Page et al., 2021).

## METHODS

### Eligibility criteria

Eligible papers were those that reported primary studies about the effects of VRn (with or without a soundscape), on the mental health and wellbeing of students in higher education (college, university). Relevant papers must have included interventions consisting of exposure to VRn, intended as reproduction (360° video) or simulation (CGI) to the natural environment through fully immersive or semi‐immersive technology. Definitions of key eligibility parameters were as follows:

#### Virtual reality

Various definitions for VR exist (Maravilla et al., 2019). For our purposes, VR is defined as an immersive human‐computer interaction derived from 3D CGI or real‐scene imagery captured by 360° camera. Fully immersive technology includes a device that produces a 360° view of virtual nature and creates the illusory perception of being enclosed within, and interacting with, a real environment including HMD, Cave Automatic Virtual Environment and immersive rooms. Semi‐immersive technology are devices that do not completely exclude the view of the external world or do not provide a 360° view of the virtual environment such as mixed‐reality devices, HMD with limited range of vision and immersive screens or displays. VRn interventions with or without auditory and/or other sensory elements were included.

#### Nature

Definitions of the natural environment vary (Smith et al., 2017). For our purposes, a broad definition of natural environment was used, including any classification and quantification of natural environment by type (green and blue spaces) or quality. Urban spaces such as a VR urban park with vegetation were included.

#### Mental health and wellbeing

We included studies of mental health and wellbeing outcomes relevant to three biopsychosocial theories (biophilia hypothesis (Kellert & Wilson, 1993, Wilson, 1984), stress recovery theory (Ulrich, 1983) and attention restoration theory (Kaplan, 1995; Kaplan & Kaplan, 1989)) of *real* nature benefits. Hence, studies of associations between VRn and constructs, such as anxiety, depression, stress, mood and cognition, were included. Both self‐reported outcome measures and physiological parameters were included.

#### Study design

Eligible papers had to contain a study design classified as experimental or quasi‐experimental, including randomised and non‐randomised controlled trials (either with parallel conditions or cross‐over design) with pre‐post or post‐assessments, as well as non‐controlled trials (experiments) with pre‐post assessments. Within the medical discipline, randomised controlled trials (RCTs) are considered the ‘gold standard’ for demonstrating causality and whether causal inferences can be made from experimental studies is contested (Gianicolo et al., 2020). In this systematic review, causal inference from experimental studies is considered acceptable, and the term ‘effect’ is used when reporting the results of experimental studies in this article.

Published pre‐printed and peer‐reviewed articles in the form of reports of original research were included. Correlational studies were excluded as were discussion articles and opinion pieces because these types of publication would not enable us to determine if VRn was associated with beneficial effects on mental health and wellbeing. No age limitation of higher education students was applied. Studies about school students were excluded. If the article was not clear on participants (i.e., it was not explicitly mentioned that they were higher education students, etc.) or outcomes, then it was excluded. Only studies written in English were included. No publication year restriction was applied.

### Information sources

The first search was conducted in May 2023 by an information specialist (CO). No publication date was imposed, but varied by database, for example, EMBASE includes articles published from 1974 and CINAHL touts indexing dating back to 1937. A number of search strategies were initially conducted to see which gave the most focused results pertaining to the aims of the systematic review without being too narrow to exclude studies. The optimum strategy was then carried out in the following eight electronic databases: OVID (Embase, MEDLINE, PsycINFO), SCOPUS, CINAHL, Cochrane Library, PubMed and GreenFILE. A re‐run search was conducted for the period May 2023 – November 2024 to include more recently published articles.

### Search strategy

Boolean operators were used, and Medical Subject Headings (MeSH) and functionality utilised where available, while still endeavouring for consistency in search terms and patterns across databases to build the search (Supporting Information).

### Selection process

Search results from each electronic database were exported to a spreadsheet and referencing management tool by one reviewer (GH). Duplications were identified electronically as well as during the manual screening process and removed (GH). Two reviewers (GH, PV) independently screened titles and abstracts of the records retrieved. Full texts of the remaining included articles were then obtained, independently reviewed, and checked for eligibility by these two researchers who resolved any disagreement by consensus. At this stage, one reviewer had included 17 articles, and a second reviewer had included 19 articles and after discussion, the additional two articles were not included. The second re‐run search repeated this process and resulted in an additional four more articles, bringing up the total number of articles to 21. Subsequently, an additional three articles identified from previously included articles' citations led to a final total of 24 papers in the systematic literature review.

### Data collection process and data items

Data from each selected article were extracted using predesigned standardised tables in spreadsheets developed by one reviewer (GH). Data extracted included: first author of study, date of publication, title of manuscript, design, aims and objectives, hypothesises, country, methods, sample size and characteristics, randomisation, interventions, primary and secondary outcomes and results. The Theory Coding Scheme (Michie & Prestwich, 2010) was used to describe the theoretical basis of interventions; it comprises 19 items and a clear description of how to code each item. All items are listed under the following six categories, which can be used to assess the use of theory: 1) Is theory/model mentioned? 2) Are the relevant theoretical constructs targeted? 3) Is theory used to select recipients or tailored interventions? 4) Are the relevant theoretical constructs measured? 5) Is theory tested? 6) Is theory refined? The Template for Intervention Description and Replication (TIDieR) was completed for each included study (Hoffmann et al., 2014). TiDieR has 12 sections to describe the intervention including theoretical underpinning for the intervention, materials and procedures (e.g., technology), who provides the intervention, mode of delivery, where it is delivered, dose (e.g., duration of virtual nature exposure), tailoring, modification and how well it is delivered and fidelity. The extraction procedure was completed independently by one reviewer (GH) and re‐checked across by another reviewer (PV), and any inconsistencies were resolved through discussion with all reviewers/authors, resulting in a consensus. Authors were not contacted if data for extraction were not reported in the included manuscript.

### Risk of bias

A critical appraisal and report of the risk of bias was assessed separately by two reviewers (PV, AB) using a modified Downs and Black checklist for randomised and non‐randomised studies (Downs & Black, 1998). Previously reported cut‐off scores were used (as per Hooper and colleagues [Hooper et al., 2008]). The quality assessments by the two reviewers were mostly aligned, and any disagreements concerning study quality/risk of bias were discussed.

### Synthesis methods

All 24 studies were included in the narrative synthesis. Two reviewers (GH, PV) summarised in narrative and tabular format: study aims, hypothesises, sample, design, experimental conditions and allocation; VR nature content (e.g., type of nature environment); subjective psychological and objective psychophysiological outcome variables and results of VRn's effect on mental health and wellbeing.

In line with recommendations provided by Cochrane Library, the extracted data would be synthesised quantitatively through a meta‐analysis, assuming that: i) all the outcomes are comparable and can be pooled meaningfully, ii) all the interventions and control/comparison conditions are reasonably similar, iii) the necessary data are available for the included studies or provided by the authors, and iv) at least two of the identified studies will present all previously described characteristics (Deeks et al., 2022). We were not able to conduct a meta‐analysis of data from studies comparing VRn versus real nature because only two studies conducted this comparison. We identified potential meta‐analyses in which VRn was compared to, i) no intervention group, and ii) 2D flat‐screen nature for the outcomes self‐reported mood and anxiety and contacted authors for missing primary data if it was not reported in the paper or supplementary material. It was decided that a meta‐analysis would not be meaningful for a comparison with the no intervention group because of the heterogeneity of interventions in the no intervention group and missing data. We decided to conduct a meta‐analysis using Comprehensive Meta‐Analysis Version 4 (Borenstein, 2022) from three studies comparing VRn versus flat‐screen nature for the outcome anxiety (data were not available for two studies). The meta‐analysis was conducted by one reviewer (PT).

## RESULTS

### Study selection

**FIGURE 1:**
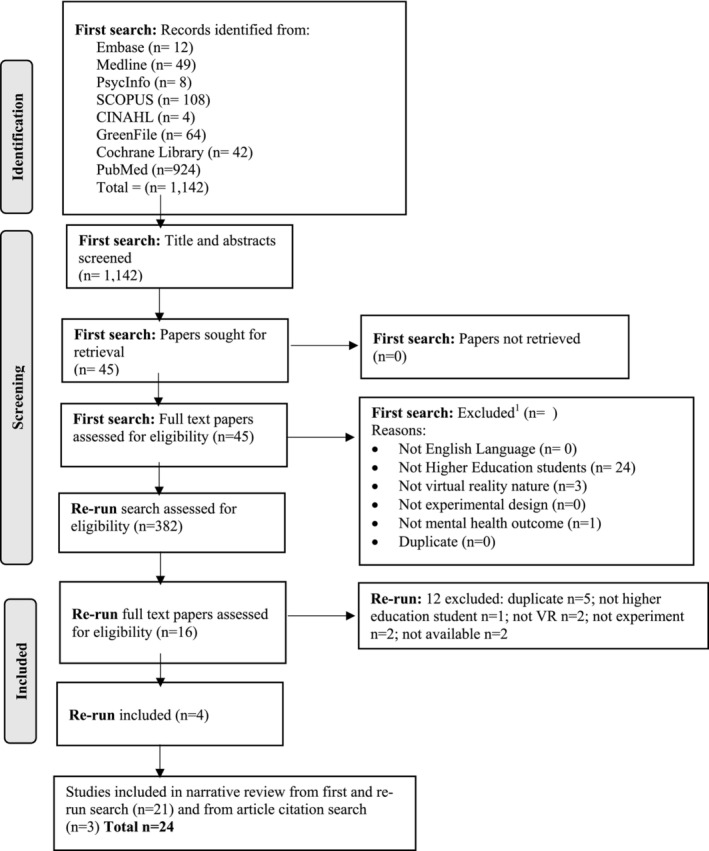
Prisma.

The search yielded a total of 1,142 hits (Figure 1). Based on screening titles and abstracts by one reviewer (GH), 45 manuscripts were assessed as potentially eligible and sought for retrieval; 17 full paper manuscripts met the eligibility criteria (Mostajeran et al., 2023, Mostajeran et al., 2021, H. Li, Dong, et al., 2021, Valtchanov et al., 2010, Zhang et al., 2021, Reece et al., 2022, Theodorou et al., 2023, Suseno & Hastjarjo, 2023, Weixin et al., 2022, Li et al., 2020, Jo et al., 2021, Léger & Mekari, 2022, Hsieh et al., 2023, Chan et al., 2023, Browning et al., 2023, Alyan et al., 2021, Browning, Mimnaugh, et al., 2020) while 23 did not. After a second reviewer (PV) independently screened papers, two papers that were initially excluded were brought forward for inclusion (H. Li, Zhang, et al., 2021, Gao et al., 2019) following a discussion by members of the review team, bringing the number of included papers to 19. During the data extraction process, two papers were excluded because one had creativity and not mental health as the main outcome (Li, Du, et al., 2022) and the other did not report outcomes for students (Zhang et al., 2021). This resulted in 17 included studies from the first search. The subsequent re‐run search identified 382 potential papers, and 4 additional papers were included, bringing the new total to 21. An additional three articles identified from included paper citations led to a final total of 24 papers.

### Study characteristics

#### Country and participants

Table 1 in the Supporting Information shows that 12 experimental studies were conducted in Asia, (Alyan et al., 2021, Chan et al., 2023, Chen et al., 2023, Gao et al., 2019, Hsieh et al., 2023, Jo et al., 2021, Li et al., 2020, H. Li, Zhang, et al., 2021, Suseno & Hastjarjo, 2023, Weixin et al., 2022, Zhao et al., 2024) eight in Europe (Martínez Manchón & Šimunić, 2024; Mostajeran et al., 2021; Mostajeran et al., 2023; O'Meara et al., 2020; Reece et al., 2022; Theodorou et al., 2023; Villani et al., 2007; Villani & Riva, 2008) and four in North America (Browning, Mimnaugh, et al., 2020, Browning et al., 2023, Léger & Mekari, 2022, Valtchanov et al., 2010). Fifteen experimental studies used a between‐groups design (usually also including within‐groups results), six studies used a within‐subjects design, one used a repeated measures design, one a randomised crossover design and one conducted a paired comparison of the post‐test measures of the two virtual environments (not the improvements of the measures compared to the baseline). The total number of participants across all studies was n = 1,419 (range of *n* was 10–189). The age range of participants was 17 to 57 years of age. Eleven studies excluded students with a current, or history of, mental illness (Alyan et al., 2021, Browning, Mimnaugh, et al., 2020, Browning et al., 2023, Chen et al., 2023, Gao et al., 2019, Léger & Mekari, 2022, O'Meara et al., 2020, You et al., 2023, Suseno & Hastjarjo, 2023, Reece et al., 2022, Hsieh et al., 2023). All studies were conducted in university laboratories.

#### Outcome variables

Tables 2 and 3 in the Supporting Information show the self‐reported and objective outcome variables (note, some measured psychological domains to describe sample characteristics but not as a mental health outcome variable) that were assessed in the 24 studies, respectively. Fourteen studies measured mood (Alyan et al., 2021, Browning, Mimnaugh, et al., 2020, Browning et al., 2023, Chan et al., 2023, Gao et al., 2019, H. Li, Zhang, et al., 2021; Martínez Manchón & Šimunić, 2024; Mostajeran et al., 2023, Mostajeran et al., 2021, O'Meara et al., 2020, Valtchanov et al., 2010, Villani et al., 2007, Villani & Riva, 2008, Zhao et al., 2024). Ten studies measured anxiety (Browning et al., 2023; Chen et al., 2023; Hsieh et al., 2023; Li et al., 2020; Mostajeran et al., 2021; O'Meara et al., 2020; Reece et al., 2022; Suseno & Hastjarjo, 2023; Villani et al., 2007; Villani & Riva, 2008). Seven studies measured stress (Martínez Manchón & Šimunić, 2024; Mostajeran et al., 2023, Mostajeran et al., 2021, Reece et al., 2022, Villani et al., 2007, Villani & Riva, 2008, You et al., 2023). Eight studies measured cognition (Gao et al., 2019, Léger & Mekari, 2022; Martínez Manchón & Šimunić, 2024; Mostajeran et al., 2023, Mostajeran et al., 2021, O'Meara et al., 2020, Valtchanov et al., 2010, You et al., 2023). Thirteen studies measured cardiovascular activity – heart rate (pulse) (HR), heart rate variability (HRV), blood volume pressure (BVP) ‐ which are markers of changes in autonomic nervous system activity (Alyan et al., 2021; Chan et al., 2023; Hsieh et al., 2023; Jo et al., 2021; Li et al., 2020; Mostajeran et al., 2021; Suseno & Hastjarjo, 2023; Valtchanov et al., 2010; Villani et al., 2007; Villani & Riva, 2008; Weixin et al., 2022; You et al., 2023; Zhao et al., 2024). Increases in HR cannot distinguish between specific emotions such as anxiety, tension, anger or pleasure and therefore self‐reports of psychological constructs, such as mood and stress are recommended for simultaneous use to facilitate interpretation of objective measurement. Ten studies used skin conductivity levels (SCL) which is the measurement of the electrical conductivity of the skin that can be used as a bio‐signal reflecting anxiety (Alyan et al., 2021, Browning, Mimnaugh, et al., 2020, Li et al., 2020, Mostajeran et al., 2021, Suseno & Hastjarjo, 2023, Valtchanov et al., 2010, Villani & Riva, 2008, You et al., 2023, Zhao et al., 2024, Villani et al., 2007). Increases in SCL can however, represent positive or negative affective arousal so self‐report measures are sometimes also simultaneously used to determine the valence. Brain activity was used to assess relaxation and attention effects. Six studies measured brain activity by Electroencephalogram (EEG), which is a physiological monitoring method that records brain activity from electrodes attached to a person's scalp (Chen et al., 2023, Gao et al., 2019, H. Li, Zhang, et al., 2021, Reece et al., 2022, Zhao et al., 2024, Jo et al., 2021). A handful of studies used some domains interchangeably for example, mood and stress (Reece et al., 2022) and in these cases, we decided which term we would use for categorisation purposes.

#### VR nature components

Below, is a summary of key VRn components. Very few of the studies (identified below) included in this review compared the effects of different VRn components. Yet, there is emerging evidence different components may have different effects which is why these are described in this paper; for example, studies have shown that items displayed in a 360^°^ video environment were more rapidly perceived compared to a CGI environment (Salamin & Hardorn, 2018) and another study found that beaches compared to green spaces cause lower breathing rates and sympathetic nervous system activity (Hooyberg et al., 2023). Table 1 in the Supporting Information shows the different experimental conditions that were compared (e.g. real outdoor forest versus 360^°^ images) and Table 4 in the Supporting Information show the VRn components for each study.

##### *Computer‐generated imagery or 360* ^°^ *image*

No studies compared CGI versus 360^°^ images; instead, studies either used CGI or 360^°^ images. One study used CGI and 360^°^ images (video or photo capture) of *real* nature, (Martínez Manchón & Šimunić, 2024) 11 studies tested CGI of nature, (Alyan et al., 2021, Chan et al., 2023, Chen et al., 2023, Li et al., 2020, H. Li, Zhang, et al., 2021, Mostajeran et al., 2023, Suseno & Hastjarjo, 2023, Valtchanov et al., 2010, Weixin et al., 2022, You et al., 2023, Zhao et al., 2024) and the other 12 studies tested 360^°^ images.

##### Type of nature content

No studies compared different types of VRn environments, for example, comparing the effects on mental health parameters of beach compared to forest environments. The most common VRn environment was forest; 12 studies tested VRn forests exclusively (Alyan et al., 2021, Browning, Mimnaugh, et al., 2020, Chan et al., 2023, Chen et al., 2023, Hsieh et al., 2023, Jo et al., 2021, Léger & Mekari, 2022, Li et al., 2020, Mostajeran et al., 2023, Mostajeran et al., 2021, O'Meara et al., 2020, Valtchanov et al., 2010).

##### Sound and other stimuli

Fourteen studies incorporated auditory stimuli, such as birdsong, flowing water, wind (Alyan et al., 2021, Browning, Mimnaugh, et al., 2020, Browning et al., 2023, Chan et al., 2023, Hsieh et al., 2023, Léger & Mekari, 2022; Martínez Manchón & Šimunić, 2024; Mostajeran et al., 2023, O'Meara et al., 2020, Reece et al., 2022, Suseno & Hastjarjo, 2023, Valtchanov et al., 2010, Villani et al., 2007, Villani & Riva, 2008). Two studies included other sensory stimuli; one study included smells of a forest via a Wick air freshener (Valtchanov et al., 2010) and another used a fan to simulate the feeling of wind (Suseno & Hastjarjo, 2023). One study compared VRn forests with no sound (control) versus low decibel and high decibel VRn forest sound (Hsieh et al., 2023). The study found that both low and high‐decibel sound VRn lowered anxiety; there were no significant differences between 360^°^ nature with no sound vs. with high decibel water sound vs. with low decibel water sound for anxiety; there were significant differences in emotional arousal with low sound levels calming emotions better than high decibel or no sound conditions; for most cardiovascular outcomes, there were no significant differences between the three experimental conditions: 360^°^ nature with no sound vs. with high decibel water sound vs. with low decibel water sound; however, there were significant differences in LF/HF and LF ratios among the three conditions, with low and high decibel conditions activating the parasympathetic nervous system (Hsieh et al., 2023). One study compared different levels of light in a VRn forest environment (Li et al., 2020). The study found CGI forest environments with day light scenes (i.e. light but not lighter and lightest) significantly reduced anxiety compared to the darkest (i.e. darkest but not darker and dark) night scenes; CGI forest environments with daylight scenes showed a significant increase in blood volume pulse than dark night scenes and CGI forest environments with daylight scenes showed a significant decrease on skin conductivity levels than dark night scenes (Li et al., 2020).

##### Interaction

Eight studies included interactive activities, such as walking, bursting bubbles, fishing and watering activities (Alyan et al., 2021, Chan et al., 2023, Léger & Mekari, 2022, H. Li, Zhang, et al., 2021; Martínez Manchón & Šimunić, 2024; Suseno & Hastjarjo, 2023, Valtchanov et al., 2010, Weixin et al., 2022). Only one study compared interactive vs. non‐interactive VRn and found that the change/decrease of stress levels and tenseness was more pronounced in the group exposed to nature interactively than the control group and group exposed to nature non‐interactively (Martínez Manchón & Šimunić, 2024).

##### Dose and exposure time

No studies compared the effects of different doses of nature or exposure time. Most studies (n = 18) tested one dose of VRn. Exposure time ranged from 1 minute and 25 seconds to 10 minutes.

##### Immersion/presence

Six studies included a self‐report measure to compare the effect of experimental conditions on sense of presence/immersion because greater sense of presence is expected to render more beneficial mental health and wellbeing effects (H. Li, Zhang, et al., 2021, Mostajeran et al., 2023, Mostajeran et al., 2021, Reece et al., 2022, Villani et al., 2007, Villani & Riva, 2008).

#### Theories and rationale for use of VR nature

Theories for intervention studies are used to guide what components are necessary to have an effect and to explain that effect. Studies referred to the three main theories of the beneficial effects of *real* nature on human health to justify studying VRn (Table 5 in the Supporting Information). Fifteen, 13 and four papers cited attention restoration theory, stress recovery theory and biophilia hypothesis, respectively. A minority of studies measured constructs that are associated with one or more of these biopsychosocial theories: restorativeness (n = 5) using for example, the Perceived Restorativeness Scale (Hartig et al., 1997), nature connectedness (n = 4) and perceptions of the environment for example, beauty and disgust in the nature (n = 2) and perceived safety, visibility, variety (n = 1).

No study justified studying the effects of VRn on the student population despite only including this group as participants. Hence, why students were the target group and why VRn was considered to have an effect on their mental health and wellbeing was not articulated. Various rationales were presented for the use of VRn to support mental health and wellbeing in general. The most common justification (n = 23) was nature's positive effects on mental health, such as reductions in aggression, depression, stress, nervousness, negative emotions, phobias, disordered eating and anxiety, and increases in self‐esteem, self‐efficacy, positive emotions, mood, restorativeness, psychological energy, life satisfaction, aliveness, vitality and psychological wellbeing. Other justifications included (n = 12) nature's effects on cognition, such as improvements in learning, problem‐solving, memory, cognitive fatigue, attention recovery and creativity. The effects of nature on physical health were less cited (n = 5) and included reductions in pain and occurrences of illness and disease such as obesity and diabetes, and improvements to the immune system and healing.

The main rationale presented for the use of VR as opposed to *real* nature was *access*, with 12 papers citing lack of access to *real* nature as a rationale for VRn. Cited examples of restrictions on access to *real* nature included urbanisation, limited expendable income, limited time, poor mobility and prolonged lockdowns during the Covid‐19 pandemic. Exemplary groups of the population with limited access to *real* nature that were cited included prison inmates, hospital patients and people living in care homes and isolated and confined environments, such as polar regions, outer space and cargo ships. Some authors (n = 5) referred to the advantages of VR for controlling the *environment*; they referred to *real* nature posing risks to human safety from physical hazards (forest fires, floods), infectious diseases (Lyme borreliosis, malaria) and the ability in VR to change weather conditions and lighting and eliminate sensory disturbances, such as traffic noise, graffiti, litter and smells in order to yield greater therapeutic effects.

### Risk of bias

The quality of the 24 studies was found to be variable, with total scores ranging from 21 to 10 out of a maximum possible score of 28 (Table 6 in the Supporting Information). None of the studies had a score above 25, which is considered ‘excellent’. Two studies were found to be of ‘good’ quality, 18 of ‘fair’ quality and four studies were assessed as being of ‘poor’ quality, with a score below 14. As Table 6 in the Supporting Information shows the six main reasons for studies being of poor quality were: distributions of confounders in each group of participants are not clearly described, there is no adequate adjustment for confounding in the analyses from which the main findings are drawn, adverse events of the intervention are not reported, participants asked to participate are not representative of the entire population from which they are recruited, participants who are prepared to participate are not representative of the entire population from which they are recruited and the staff places and facilities where participants are located are not representative of the majority for that population.

### Results of synthesis

Table 1 below shows which studies reported that VRn had a beneficial effect on outcomes. Tables 7 and 8 in the Supporting Information present the outcomes for each study.

**TABLE 1:** Beneficial effects on outcomes

	Self‐reported						
Author	Mood	Anxiety	Stress	Cognition	Cardiovascular	Skin conductivity	Brain activity
Alyan 2021	✔︎	‐	‐	‐	✔︎	✔︎	‐
Browning 2020	✔︎	‐	‐	‐	‐	✔︎	‐
Browning 2023	✔︎	✔︎	‐	‐	‐	‐	‐
Chan 2023	✔︎	‐	‐	‐	✔︎	‐	‐
Chen 2023	‐	✔︎	‐	‐	‐	‐	✔︎
Gao 2019	✔︎	‐	‐	✔︎	‐	‐	✔︎
Hsieh 2023	‐	✔︎	‐	‐	✔︎	‐	‐
Jo 2021	‐	‐	‐	‐	✔︎	‐	✔︎
Leger 2022	‐	‐	‐	✔︎	‐	‐	‐
Li 2020	‐	✔︎	‐	‐	✔︎	✔︎	
Li 2021	✔︎	‐	‐	‐		‐	✔︎
Manchon 2023	✔︎	‐	✔︎	✗	‐	‐	‐
Mostajeran 2021	✔︎	✗	✗	✔︎	✔︎	✗	‐
Mostajeran 2023	✔︎	‐	✔︎	✔︎	‐	‐	‐
O’Meara 2020	✔︎	✔︎	‐	✗	‐	‐	‐
Reece 2022	‐	✔︎	✔︎	‐	‐	‐	✔︎
Weixin 2023	‐	‐	‐	‐	✔︎	‐	‐
Suseno 2023	‐	✔︎	‐	‐	✔︎	✔︎	‐
Theodorou 2023	‐	‐	‐	‐	‐	‐	‐
Valtchanov 2010	✔︎	‐	‐	✗	✔︎	✔︎	‐
Villani 2007	Not reported	✔︎	✔︎	‐	✗	✗	‐
Villani 2008	✔︎	✔︎	✔︎	‐	✗	✗	‐
You 2023	‐	‐	✔︎	✗	✗	✗	‐
Zhao 2024	✔︎	‐	‐	‐	✗	✔︎	‐

Study did not measure this outcome

✔︎ VRn had a beneficial effect on this outcome variable

✗ VRn did not have a beneficial effect on this outcome variable

#### Mental health and wellbeing outcomes

Thirteen out of 14 studies that assessed mood by self‐report show that VRn had some beneficial pre‐post intervention effect on mood (Alyan et al., 2021, Browning, Mimnaugh, et al., 2020, Chan et al., 2023, H. Li, Zhang, et al., 2021, Gao et al., 2019, Mostajeran et al., 2021, Zhao et al., 2024, O'Meara et al., 2020, Villani & Riva, 2008, Valtchanov et al., 2010, Mostajeran et al., 2023, Browning et al., 2023; Martínez Manchón & Šimunić, 2024) and one study did not report results (Villani et al., 2007). Nine (Browning et al., 2023; Chen et al., 2023; Hsieh et al., 2023; Li et al., 2020; O'Meara et al., 2020; Reece et al., 2022; Suseno & Hastjarjo, 2023; Villani et al., 2007; Villani & Riva, 2008) out of 10 studies that assessed anxiety by self‐report show that VRn lowered anxiety and one did not (Mostajeran et al., 2021). Six (Martínez Manchón & Šimunić, 2024; Mostajeran et al., 2023; Villani et al., 2007, You et al., 2023, Villani & Riva, 2008, Reece et al., 2022) out of the seven studies that assessed stress by self‐report show that VRn reduced stress and one did not (Mostajeran et al., 2021). Four (Gao et al., 2019; Léger & Mekari, 2022; Mostajeran et al., 2021; Mostajeran et al., 2023) out of the eight studies that assessed cognition by self‐report show that VRn restored cognition and four did not (Martínez Manchón & Šimunić, 2024; O'Meara et al., 2020; Valtchanov et al., 2010, You et al., 2023). Nine (Alyan et al., 2021; Chan et al., 2023; Hsieh et al., 2023; Jo et al., 2021; Li et al., 2020; Mostajeran et al., 2021; Suseno & Hastjarjo, 2023; Valtchanov et al., 2010; Weixin et al., 2022) out of the 13 studies that assessed cardiovascular effects show that VRn had a beneficial effect and four did not (Villani et al., 2007; Villani & Riva, 2008; You et al., 2023; Zhao et al., 2024). Six (Alyan et al., 2021, Browning, Mimnaugh, et al., 2020, Li et al., 2020, Suseno & Hastjarjo, 2023, Valtchanov et al., 2010, Zhao et al., 2024) out of the 10 studies that assessed skin conductivity levels show that VRn had a beneficial effect and four did not (Mostajeran et al., 2021; Villani et al., 2007; Villani & Riva, 2008; You et al., 2023). All five studies that measured brain activity show changes suggesting that VRn had a beneficial effect on attention and engagement (Chen et al., 2023, Gao et al., 2019, Jo et al., 2021, H. Li, Zhang, et al., 2021, Reece et al., 2022).

#### VRn vs. real nature

Only two studies compared exposure to VRn vs. real nature. One study that measured the effect on cognition (executive functioning and memory functioning) found that both a VRn walk and *real* nature walk improved cognition, and there was no significant difference between these two conditions (Léger & Mekari, 2022). One study that measured the effect on mood found that VRn did not improve positive affect whereas *real* outdoor nature did, both conditions reduced negative affect, and there was no significant difference in reductions (Browning, Mimnaugh, et al., 2020). This study also measured SCL and found that VRn and *real* nature reduced stress measured by SCL, and there was no significant difference between these two conditions (Browning et al., 2020a).

#### VRn vs. no intervention group

Six studies compared exposure to VRn vs. no intervention group (Browning, Mimnaugh, et al., 2020, Browning et al., 2023, Jo et al., 2021; Martínez Manchón & Šimunić, 2024; Mostajeran et al., 2021, Villani et al., 2007). One of these studies did not report results of the comparison of VRn with the no intervention group, although a no intervention group was included (Mostajeran et al., 2021). Four studies reported on mood. One study found that VRn preserved positive affect whereas the no intervention group reduced positive affect, and both VRn and the no intervention group reduced negative affect (Browning, Mimnaugh, et al., 2020). The study also found that VRn tended to show higher SCL levels and a more steadily increasing slope compared to the no intervention group. When interpreted alongside mood scores, the authors concluded that SCL scores were indicative of the maintenance of positive affect (Browning, Mimnaugh, et al., 2020). Two studies found that VRn had a greater beneficial effect on mood compared to the no intervention group (Browning et al., 2023; Martínez Manchón & Šimunić, 2024). One study did not find significant differences between VRn and no intervention group for mood or skin conductivity (Villani et al., 2007). Two studies reported on anxiety. One study found that VRn reduced anxiety significantly more than the no intervention group (Browning et al., 2023), and one study did not find any significant difference (Villani et al., 2007). One study reported that VRn reduced stress significantly more than the no intervention group (Martínez Manchón & Šimunić, 2024), and one study did not (Villani et al., 2007). One study did not find any significant difference between VRn and no intervention group for cognition (executive functioning and memory functioning) (Martínez Manchón & Šimunić, 2024). One study found that VRn had a greater beneficial effect on heart rate than the no intervention group (Jo et al., 2021), and one study did not find any difference (Villani et al., 2007). One study concluded that EEG measures indicated that immersion and concentration were significantly higher for VRn compared to the no intervention group (Jo et al., 2021).

#### VRn vs. flat‐screen nature

**FIGURE 2:**
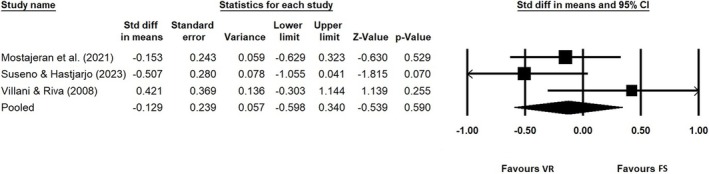
Meta‐analysis comparing VR nature and flat‐screen nature for three studies with anxiety as an outcome.

Seven studies compared exposure to VRn vs. flat‐screen nature (Jo et al., 2021; Mostajeran et al., 2021; Reece et al., 2022; Suseno & Hastjarjo, 2023; Valtchanov et al., 2010; Villani et al., 2007; Villani & Riva, 2008). All five studies that measured anxiety found no significant difference (Mostajeran et al., 2021; Reece et al., 2022; Suseno & Hastjarjo, 2023; Villani et al., 2007; Villani & Riva, 2008). Figure 2 presents the forest plot with standardised difference in means calculated using Hedges' ‘g’ and 95% confidence intervals for three studies with anxiety as an outcome where primary data were available. The random‐effects model pooled estimate was −0.598 to 0.340. The heterogeneity observed was moderately high I^2^ = 50, although this should be interpreted with great caution as only a small sample (N = 147) was included in the meta‐analysis.

Three studies that measured mood found no significant difference (Mostajeran et al., 2021; Villani et al., 2007; Villani & Riva, 2008) and one study found no significant difference in reductions of negative affect, but VRn increased positive affect more than a slideshow of abstract paintings of colours found in nature (Valtchanov et al., 2010). All four studies that measured stress found no significant difference (Mostajeran et al., 2021; Reece et al., 2022; Villani et al., 2007; Villani & Riva, 2008). The two studies that measured cognition (executive functioning and memory functioning and mental arithmetic) found no significant difference (Mostajeran et al., 2021; Valtchanov et al., 2010). Four of the five studies that measured heart rate found no significant difference (Suseno & Hastjarjo, 2023; Valtchanov et al., 2010; Villani et al., 2007; Villani & Riva, 2008), and one study reported that VRn had a more beneficial effect on heart rate (Jo et al., 2021). Four of the five studies that measured skin conductance found no significant difference (Mostajeran et al., 2021; Suseno & Hastjarjo, 2023; Villani et al., 2007; Villani & Riva, 2008), and one study found VRn decreased skin conductance more than a slideshow of abstract paintings of colours found in nature (Valtchanov et al., 2010). Both studies that measured brain activity concluded that EEG scores indicate that immersion, concentration and relaxation was higher in VRn compared to flat‐screen nature exposure (Jo et al., 2021; Reece et al., 2022).

#### Sense of presence, restorativeness, nature connectedness, perceptions of environment

All six studies that included an assessment of presence suggest that VRn influenced sense of presence (Mostajeran et al., 2021, Mostajeran et al., 2023, Reece et al., 2022, H. Li, Zhang, et al., 2021, Villani et al., 2007, Villani & Riva, 2008). All five studies that assessed restorativeness show that VRn influenced restorativeness (Browning, Mimnaugh, et al., 2020, Mostajeran et al., 2023, H. Li, Zhang, et al., 2021Theodorou et al., 2023, Zhao et al., 2024). Three studies did not find that nature connectedness was a significant covariate in any of the analyses (Chan et al., 2023; Martínez Manchón & Šimunić, 2024; O'Meara et al., 2020) and one study found that nature connectedness was higher in the nature environments compared to the non‐biophilic environment (You et al., 2023). Two studies found that perceptions of beauty influenced mental health and wellbeing outcomes (Browning et al., 2023, Browning, Mimnaugh, et al., 2020) and one study found that feelings of safety were significantly higher in VRn daylight compared to darkness (Li et al., 2020).

## DISCUSSION & CONCLUSION

United Nation's SDGs and ‘One Health’ approaches call for much closer attention being paid to the inter‐relationships and impacts of real or simulated natural environments on human health and the impact of using real nature and manufacturing VRn on the natural environment. Claiming that VRn is preferable or equal to real nature is problematic. VRn potentially provides health benefits to humans without exploiting and negatively impacting real environments such as air, water and soil quality (Wu et al., 2021). On the other hand, the manufacture of digital technology has a negative environmental impact (Khakpour et al., 2020), although VR manufacturing companies may be able to reduce environmental damage (Chen et al., 2021; Litleskare et al., 2020). There is a potential negative effect on climate change due to the amount of energy used by individuals wearing HMDs to experience VR. This environmental cost may be offset if VR improves people's knowledge and behaviours to protect the environment. VR for instance, has been used to promote improvements in knowledge about the detrimental efforts on the planet from climate change (Tisoglu et al., 2025). VRn could motivate people to engage in actions outdoors that protect and sustain real nature, particularly if behavioural and motivational components are embedded, but not if it increases screen‐time at the expense of engaging in pro‐environmental behaviours (Spangenberger et al., 2024). It could substitute in‐person treatment delivered by mental health services at a health centre to reduce carbon footprint and in doing so, contribute towards a more sustainable planet but not if this is at the expense of providing sub‐optimal support for people with mental health conditions. Finally, the different impacts of real nature and VRn on mental health also must be taken into consideration given that a meta‐analysis of six studies with samples consisting primarily of young adults with a pooled age of 24, found that exposure to real nature has a significantly stronger effect on improving mood than virtual nature experiences (Browning, Shipley, et al., 2020).

Twenty‐four studies of VRn were included in this systematic literature review. Studies assessed mental health symptomology including mood, anxiety, stress and cognition by self‐report and objective physiological measures (HR, SCL, EEG). Studies that were included in this review show mental health benefits immediately after a VRn experience for higher education students. Despite the review indicating that there are mental health benefits after VRn exposure, there are several reasons why we are extremely cautious when drawing conclusions about relationships between VRn and mental health for higher education students from this body of work. First, all the studies included in this review assessed the relationship between VRn and mental health under laboratory test conditions, which means that we do not know yet if benefits can be achieved in real‐world contexts. The effect of VRn in situ might be quite different to both laboratory conditions. Second, the quality of studies is sub‐optimal. Sample sizes were often small with insufficient statistical power, and drawing conclusions from studies with a total sample of *n* = 1,419 participants drawn from different countries must be done with caution. Third, it is not possible to collectively synthesise the results of all these experimental studies because they are not comparing like with like. Studies had different aims and comparator experimental groups (for example, real nature vs. 360^°^ nature, daylight vs. nightlight CGI, 360^°^ vs. 2D slideshow). Furthermore, studies tested different VRn environments, sensory stimuli, dose, exposure time and outcomes. How meaningful it is to summarise the effect of VRn on mental health is therefore debatable. Hence, like other research teams who have conducted systematic reviews of VRn, we found that it was challenging to make comparisons among research studies, and there were also inconsistencies in results (Frost et al., 2022).

The review highlights several gaps in the literature corpus. First, the most studied VRn is a forest environment, which means that there is very limited empirical evidence regarding other types of nature environments, such as urban green spaces and blue spaces. Second, the review shows that current knowledge about the required exposure time necessary to improve mental health and wellbeing is limited since the majority of studies did not compare different frequencies and doses of exposure. Third, these studies included higher education students and therefore, the findings can only be applied to this group of the population. The reasons for only including higher education students as participants were not given in the included studies; instead, the authors provided general rationales for VRn, such as nature's benefits to mental health and restrictions on access to real nature. This means that the case for VRn and researching the benefits of VRn for specific population groups, such as higher education students, seems to be currently unarticulated. Giving a robust rationale for VRn for specific groups may have merit, particularly given that we found that other types of nature, such as 2D nature, may be just as effective and may be more accessible. Fourth, only a handful of studies measured theoretically‐related constructs of nature, such as restorativeness, nature connectedness and perceptions of beauty or disgust in nature to find out if these psychological constructs moderated or mediated observed relationships between VRn and mental health and wellbeing outcomes. This means that the mechanisms of VRn have not been thoroughly investigated, and hence, our understanding of how to target specific pathways through the design and delivery of VRn remains limited. Future research should therefore include these constructs so that we can explain the underlying mechanisms and develop VRn interventions with key active ingredients that can unlock the benefits to human health. This is particularly important given that the pathways for VRn may not be the same as for *real* nature, for example, some pathways for *real* nature may be direct, such as airborne phytoncides and negative ions that may not be available in simulated natural environments or might have a different effect (Kuo, 2015). However, as other systematic reviews highlight ‐ these limitations of current knowledge are also evident for real nature (Cleary et al., 2017; Jimenez et al., 2021).

Nature connectedness is a psychological construct that warrants further investigation. It moves beyond simply quantifying the number of contacts with nature towards evaluating an individual's sense of their relationship with the natural world. It is a construct that has been found to effectively mitigate the adverse mental health effects of global crises (wars, climate change and radioactive water contamination) in young people (Lau et al., 2024). Some studies have shown a relationship between real nature contact, nature connectedness and pro‐environmental behaviour (Liu et al., 2022), and a meta‐analysis found a small, significant causal effect of nature connection on pro‐environmental behaviour (Mackay & Schmitt, 2019). It is unclear if VRn exerts the same pro‐environmental behaviour effects as real nature mediated by nature connectedness. If VRn improves nature connectedness then it could potentially be used as an intervention to improve human relationships with the natural world in addition to real nature (Litleskare et al., 2020). More research on this subject is warranted. A systematic review and meta‐analysis of VRn on nature connectedness of six papers (nine randomised controlled trials; n = 730 participants) in which VRn was compared to, (i) non‐immersive virtual nature, (ii) immersive virtual built environments, (iii) non‐immersive virtual built environments and (iv) real nature, found a statistically significant overall effect for the first and fourth group with the former in favour of VRn and the latter in favour of real nature (Brambilla et al., 2024). Another important construct that warrants further investigation is sense of presence/immersion. VRn is claimed to offer a more immersive experience than 2D nature (de Kort et al., 2006) and therefore is expected to yield more beneficial effects on human health. This review, however, suggests that there are no significant differences between VRn and flat‐screen nature. Other studies have found a relationship between immersion and nature connectedness; in a small study (n = 28) comparing 360^°^ vs. 2D, the researchers found that irrespective of condition allocation, the more immersed participants felt in their experience, the greater they reported increased levels of nature connection but it did not translate into a stronger experimental effect of the VRn condition on nature relatedness (Spangenberger et al., 2022). Presence/immersion may be important for attention restoration because nature exposure will only result in restoration if there is sustained attention. A systematic review and meta‐analysis addressed the question of how much immersion in simulated nature is enough to yield restorative effects (Li, Ding, et al., 2022). The review was restricted to audio‐visual simulated nature experiments with immersion levels classified by projection devices and motion capture. For positive affect, medium immersion was observed to produce a larger effect than low and high immersion, but immersion level did not explain heterogeneity for negative affect (Li, Ding, et al., 2022). The authors speculate that high immersion may make people feel uncomfortable, especially if they experience cybersickness and that a highly immersive VRn experience may not be desirable for people with low technology capability. Hence, they call for more attention to the technical features of VRn, such as more technical details related to immersion (e.g. screen size, field‐of‐view) and their mechanisms of action to elucidate the association between VRn, immersion and health. However, we found that only a handful of studies compared the effect of different experimental conditions on presence/immersion.

### Limitations

VR is a rapidly evolving technology, and this systematic review included 24 unique studies about VRn and mental health and wellbeing of higher education students. Strengths of this review include the following: we combined the bibliographic records of eight large databases, the search, data extraction and synthesis processes are transparent and reproducible, and we included studies with any type of mental health and wellbeing outcome in acknowledgement that it is a profoundly heterogeneous term with multiple definitions. Limitations of this review include the following: we restricted our review to higher education students which means that the findings can only be applied to this group of the population, we did not include grey literature which may result in missing unpublished data, we did not exclude studies based on low quality hence, all of the findings need to be interpreted with caution. Beyond our control is the heterogeneity of studies, which meant that we could not easily make comparisons between research studies.

### Future research

This review highlights key gaps in knowledge that could be addressed in future research about VRn. This is not a definitive list of recommendations, but future research could include comparisons of different types of VRn environments, exposures (frequency and dose), levels of interactive activities in the VRn, sensory stimuli (especially soundscapes) and theoretically‐informed nature constructs, such as nature connectedness and restorativeness to advance understandings of the underlying pathways and mechanisms. Standardised definitions, clearly articulated rationales for VRn for specific groups of the population, agreed core VRn technical features, and a core outcome set would contribute towards overcoming the challenges of comparing between research studies. There are other lines of enquiry that deserve attention, including the use of VRn as a potential technology to reduce health inequalities that arise from some people being poorly served by access to mental health services. Of course, VRn should not be at the expense of reducing in‐person contact with practitioners if that is the preferred and more effective form of treatment. The growth of VRn coincides with concerns about increased screen‐time, use of smart devices and online learning, which have been linked to stress‐induced symptoms and poorer academic performance (Mheidly et al., 2020; Oducado & Estoque, 2021), and future studies should therefore include measures of potential negative effects. Alongside evaluating potential negative effects on human health, future studies may include evaluating the impact of VRn on the natural environment.

### Implications for practice and policy

A major advantage of VRn over real nature is that it can make nature (albeit in virtual form) more accessible to people who have access to this technology. If VRn can improve mental health and wellbeing, then it is hoped that HMD devices could be made readily available in university libraries, halls of residences, cafes, etc. However, research of this relatively novel and evolving technology is emergent, and there are significant gaps in knowledge about VRn as an intervention for higher education students. Research must investigate both potential positive (e.g., improved mood) and negative effects (e.g., discouraging real‐life nature engagement by promoting increased screen‐time). This means that there is currently a limited evidence‐base upon which to draw any firm conclusions. That said, the review suggests that nature replicated in VR shows promise for short‐term benefits to higher education students. Hence, given the current limitations in evidence, it may be considered a supplement to real nature and 2D nature rather than a substitute, especially for people who may have limited opportunities or willingness to enjoy real nature. Similar to conclusions drawn by other systematic reviews of VRn (Browning et al., 2021; Frost et al., 2022, Li, Ding, et al., 2022), continued research in this area is a worthwhile pursuit.

## CONFLICT OF INTEREST STATEMENT

None declared.

## ETHICS APPROVAL

NA

## INFORMED CONSENT

NA

## PERMISSION TO REPRODUCE MATERIAL FROM OTHER SOURCES

NA

## CLINICAL TRIAL REGISTRATION

NA

## Supporting information


**Table S1:** Title, aim, hypothesises, country, design, experimental conditions and allocation
Table S2 Subjective psychological outcomes
Table S3: Objective physiological outcomes
Table S4: VR nature components
Table S5: Rationale for VR nature and theories
Table S6: Quality Assessment Table
Table S7: Effect on mental health outcomes
Table S8: Effect on presence, restorativeness, nature connectedness, perceptions of environment

## Data Availability

Template data collection forms are available on request from the corresponding author.
