# Supplementary material for: A systematic literature review and meta‐analysis of virtual reality nature effects on higher education students' mental health and wellbeing
Source: Appl Psychol Health Well Being. 2025 Sep 4;17(5):e70060. doi: 10.1111/aphw.70060 (PMC12411694; doi:10.1111/aphw.70060)
Supplement: Supplementary file 1 — Table S1: Title, aim, hypothesises, country, design, experimental conditions and allocation Table S2 Subjective psychological outcomes Table S3: Objective physiological outcomes Table S4: VR nature components Table S5: Rationale for VR nature and theories Table S6: Quality Assessment Table Table S7: Effect on mental health outcomes Table S8: Effect on presence, restorativeness, nature connectedness, perceptions of environment [file APHW-17-0-s001.docx]

**CINAHL Search Pattern (33 Results):**

| **#** | **Query** | **Limiters/Expanders** | **Last Run Via** | **Results** |
| --- | --- | --- | --- | --- |
| S32 | S10 AND S16 AND S26 AND S31 | Expanders - Apply equivalent subjects  Search modes - Boolean/Phrase | Interface - EBSCOhost Research Databases  Search Screen - Advanced Search  Database - CINAHL Complete | 33 |
| S31 | S27 OR S28 OR S29 OR S30 | Expanders - Apply equivalent subjects  Search modes - Boolean/Phrase | Interface - EBSCOhost Research Databases  Search Screen - Advanced Search  Database - CINAHL Complete | 125,277 |
| S30 | (MH "Colleges and Universities+") | Expanders - Apply equivalent subjects  Search modes - Boolean/Phrase | Interface - EBSCOhost Research Databases  Search Screen - Advanced Search  Database - CINAHL Complete | 51,536 |
| S29 | student AND (college* OR universit* OR higher educat*) | Expanders - Apply equivalent subjects  Search modes - Boolean/Phrase | Interface - EBSCOhost Research Databases  Search Screen - Advanced Search  Database - CINAHL Complete | 83,052 |
| S28 | (MH "Student Health Services+") | Expanders - Apply equivalent subjects  Search modes - Boolean/Phrase | Interface - EBSCOhost Research Databases  Search Screen - Advanced Search  Database - CINAHL Complete | 1,636 |
| S27 | ""Higher education"" | Expanders - Apply equivalent subjects  Search modes - Boolean/Phrase | Interface - EBSCOhost Research Databases  Search Screen - Basic Search  Database - CINAHL Complete | 15,080 |
| S26 | S17 OR S18 OR S19 OR S20 OR S21 OR S22 OR S23 OR S24 OR S25 | Expanders - Apply equivalent subjects  Search modes - Boolean/Phrase | Interface - EBSCOhost Research Databases  Search Screen - Basic Search  Database - CINAHL Complete | 541,724 |
| S25 | (MH "Stress+") OR (MH "Stress, Psychological+") OR (MM "Stress Management") | Expanders - Apply equivalent subjects  Search modes - Boolean/Phrase | Interface - EBSCOhost Research Databases  Search Screen - Basic Search  Database - CINAHL Complete | 117,436 |
| S24 | (MH "Anxiety Disorders+") OR (MH "Anxiety+") OR "anxiet*" | Expanders - Apply equivalent subjects  Search modes - Boolean/Phrase | Interface - EBSCOhost Research Databases  Search Screen - Basic Search  Database - CINAHL Complete | 156,532 |
| S23 | (MH "Depression+") OR "depressi*" | Expanders - Apply equivalent subjects  Search modes - Boolean/Phrase | Interface - EBSCOhost Research Databases  Search Screen - Basic Search  Database - CINAHL Complete | 214,977 |
| S22 | "(cognit* OR affect*) AND (health* OR disorder*)" | Expanders - Apply equivalent subjects  Search modes - Boolean/Phrase | Interface - EBSCOhost Research Databases  Search Screen - Advanced Search  Database - CINAHL Complete | 0 |
| S21 | "mental* AND restor*" | Expanders - Apply equivalent subjects  Search modes - Boolean/Phrase | Interface - EBSCOhost Research Databases  Search Screen - Advanced Search  Database - CINAHL Complete | 5 |
| S20 | "mental disorder*" AND (prevent* OR control*) | Expanders - Apply equivalent subjects  Search modes - Boolean/Phrase | Interface - EBSCOhost Research Databases  Search Screen - Advanced Search  Database - CINAHL Complete | 18,923 |
| S19 | mental AND (problem* OR health difficult*) | Expanders - Apply equivalent subjects  Search modes - Boolean/Phrase | Interface - EBSCOhost Research Databases  Search Screen - Advanced Search  Database - CINAHL Complete | 43,864 |
| S18 | (MH "Research, Mental Health") OR "research, mental health" | Expanders - Apply equivalent subjects  Search modes - Boolean/Phrase | Interface - EBSCOhost Research Databases  Search Screen - Advanced Search  Database - CINAHL Complete | 2,166 |
| S17 | (MH "Mental Health") OR "mental health" | Expanders - Apply equivalent subjects  Search modes - Boolean/Phrase | Interface - EBSCOhost Research Databases  Search Screen - Advanced Search  Database - CINAHL Complete | 183,845 |
| S16 | S11 OR S12 OR S13 OR S14 OR S15 | Expanders - Apply equivalent subjects  Search modes - Boolean/Phrase | Interface - EBSCOhost Research Databases  Search Screen - Advanced Search  Database - CINAHL Complete | 549,909 |
| S15 | landscape* OR forest* OR coast* OR ocean* or sea* | Expanders - Apply equivalent subjects  Search modes - Boolean/Phrase | Interface - EBSCOhost Research Databases  Search Screen - Advanced Search  Database - CINAHL Complete | 321,342 |
| S14 | Outdoor* | Expanders - Apply equivalent subjects  Search modes - Boolean/Phrase | Interface - EBSCOhost Research Databases  Search Screen - Advanced Search  Database - CINAHL Complete | 7,139 |
| S13 | (blue OR green) AND (health OR nature OR space) | Expanders - Apply equivalent subjects  Search modes - Boolean/Phrase | Interface - EBSCOhost Research Databases  Search Screen - Advanced Search  Database - CINAHL Complete | 16,047 |
| S12 | (MH "Ecosystem+") | Expanders - Apply equivalent subjects  Search modes - Boolean/Phrase | Interface - EBSCOhost Research Databases  Search Screen - Advanced Search  Database - CINAHL Complete | 10,181 |
| S11 | (MH "Natural Environment+") OR "natur*" | Expanders - Apply equivalent subjects  Search modes - Boolean/Phrase | Interface - EBSCOhost Research Databases  Search Screen - Advanced Search  Database - CINAHL Complete | 242,109 |
| S10 | S1 OR S2 OR S3 OR S4 OR S5 OR S6 OR S7 OR S8 OR S9 | Expanders - Apply equivalent subjects  Search modes - Boolean/Phrase | Interface - EBSCOhost Research Databases  Search Screen - Advanced Search  Database - CINAHL Complete | 94,506 |
| S9 | (MH "Video-Assisted Techniques and Procedures+") | Expanders - Apply equivalent subjects  Search modes - Boolean/Phrase | Interface - EBSCOhost Research Databases  Search Screen - Advanced Search  Database - CINAHL Complete | 651 |
| S8 | 360° video* | Expanders - Apply equivalent subjects  Search modes - Boolean/Phrase | Interface - EBSCOhost Research Databases  Search Screen - Advanced Search  Database - CINAHL Complete | 30 |
| S7 | internet*base* interven* | Expanders - Apply equivalent subjects  Search modes - Boolean/Phrase | Interface - EBSCOhost Research Databases  Search Screen - Advanced Search  Database - CINAHL Complete | 2 |
| S6 | (MH "Webcasts+") OR "Streaming" | Expanders - Apply equivalent subjects  Search modes - Boolean/Phrase | Interface - EBSCOhost Research Databases  Search Screen - Advanced Search  Database - CINAHL Complete | 3,660 |
| S5 | Simulat* natur* | Expanders - Apply equivalent subjects  Search modes - Boolean/Phrase | Interface - EBSCOhost Research Databases  Search Screen - Advanced Search  Database - CINAHL Complete | 217 |
| S4 | (MH "Simulations+") OR "Simulation*" OR (MH "Computer Simulation+") | Expanders - Apply equivalent subjects  Search modes - Boolean/Phrase | Interface - EBSCOhost Research Databases  Search Screen - Advanced Search  Database - CINAHL Complete | 76,186 |
| S3 | (Virtual experiment* environment* OR VEE*) | Expanders - Apply equivalent subjects  Search modes - Boolean/Phrase | Interface - EBSCOhost Research Databases  Search Screen - Advanced Search  Database - CINAHL Complete | 8,807 |
| S2 | "(Virtual health room* OR VHR)" | Expanders - Apply equivalent subjects  Search modes - Boolean/Phrase | Interface - EBSCOhost Research Databases  Search Screen - Advanced Search  Database - CINAHL Complete | 0 |
| S1 | (MH "Virtual Realit*+") OR "Virtual reality" OR "VR" OR (MM "Virtual Reality Exposure Therapy") | Expanders - Apply equivalent subjects  Search modes - Boolean/Phrase | Interface - EBSCOhost Research Databases  Search Screen - Advanced Search  Database - CINAHL Complete | 13,739 |

Bottom of Form

**Medline Search Pattern (141 results):**

| **#** | **Query** | **Limiters/Expanders** | **Last Run Via** | **Results** |
| --- | --- | --- | --- | --- |
| S32 | S10 AND S16 AND S26 AND S31 | Expanders - Apply equivalent subjects  Search modes - Boolean/Phrase | Interface - EBSCOhost Research Databases  Search Screen - Basic Search  Database - MEDLINE with Full Text | 141 |
| S31 | S27 OR S28 OR S29 OR S30 | Expanders - Apply equivalent subjects  Search modes - Boolean/Phrase | Interface - EBSCOhost Research Databases  Search Screen - Basic Search  Database - MEDLINE with Full Text | 392,659 |
| S30 | (MH "Colleges and Universities+") | Expanders - Apply equivalent subjects  Search modes - Boolean/Phrase | Interface - EBSCOhost Research Databases  Search Screen - Basic Search  Database - MEDLINE with Full Text | 0 |
| S29 | student AND (college* OR universit* OR higher educat*) | Expanders - Apply equivalent subjects  Search modes - Boolean/Phrase | Interface - EBSCOhost Research Databases  Search Screen - Basic Search  Database - MEDLINE with Full Text | 347,894 |
| S28 | (MH "Student Health Services+") | Expanders - Apply equivalent subjects  Search modes - Boolean/Phrase | Interface - EBSCOhost Research Databases  Search Screen - Basic Search  Database - MEDLINE with Full Text | 3,197 |
| S27 | ""Higher education"" | Expanders - Apply equivalent subjects  Search modes - Boolean/Phrase | Interface - EBSCOhost Research Databases  Search Screen - Basic Search  Database - MEDLINE with Full Text | 52,844 |
| S26 | S17 OR S18 OR S19 OR S20 OR S21 OR S22 OR S23 OR S24 OR S25 | Expanders - Apply equivalent subjects  Search modes - Boolean/Phrase | Interface - EBSCOhost Research Databases  Search Screen - Basic Search  Database - MEDLINE with Full Text | 1,208,768 |
| S25 | (MH "Stress+") OR (MH "Stress, Psychological+") OR (MM "Stress Management") | Expanders - Apply equivalent subjects  Search modes - Boolean/Phrase | Interface - EBSCOhost Research Databases  Search Screen - Basic Search  Database - MEDLINE with Full Text | 151,950 |
| S24 | (MH "Anxiety Disorders+") OR (MH "Anxiety+") OR "anxiet*" | Expanders - Apply equivalent subjects  Search modes - Boolean/Phrase | Interface - EBSCOhost Research Databases  Search Screen - Basic Search  Database - MEDLINE with Full Text | 350,989 |
| S23 | (MH "Depression+") OR "depressi*" | Expanders - Apply equivalent subjects  Search modes - Boolean/Phrase | Interface - EBSCOhost Research Databases  Search Screen - Basic Search  Database - MEDLINE with Full Text | 562,507 |
| S22 | "(cognit* OR affect*) AND (health* OR disorder*)" | Expanders - Apply equivalent subjects  Search modes - Boolean/Phrase | Interface - EBSCOhost Research Databases  Search Screen - Basic Search  Database - MEDLINE with Full Text | 0 |
| S21 | "mental* AND restor*" | Expanders - Apply equivalent subjects  Search modes - Boolean/Phrase | Interface - EBSCOhost Research Databases  Search Screen - Basic Search  Database - MEDLINE with Full Text | 7 |
| S20 | "mental disorder*" AND (prevent* OR control*) | Expanders - Apply equivalent subjects  Search modes - Boolean/Phrase | Interface - EBSCOhost Research Databases  Search Screen - Basic Search  Database - MEDLINE with Full Text | 51,323 |
| S19 | mental AND (problem* OR health difficult*) | Expanders - Apply equivalent subjects  Search modes - Boolean/Phrase | Interface - EBSCOhost Research Databases  Search Screen - Basic Search  Database - MEDLINE with Full Text | 105,268 |
| S18 | (MH "Research, Mental Health") OR "research, mental health" | Expanders - Apply equivalent subjects  Search modes - Boolean/Phrase | Interface - EBSCOhost Research Databases  Search Screen - Basic Search  Database - MEDLINE with Full Text | 327 |
| S17 | (MH "Mental Health") OR "mental health" | Expanders - Apply equivalent subjects  Search modes - Boolean/Phrase | Interface - EBSCOhost Research Databases  Search Screen - Basic Search  Database - MEDLINE with Full Text | 409,903 |
| S16 | S11 OR S12 OR S13 OR S14 OR S15 | Expanders - Apply equivalent subjects  Search modes - Boolean/Phrase | Interface - EBSCOhost Research Databases  Search Screen - Basic Search  Database - MEDLINE with Full Text | 3,877,679 |
| S15 | landscape* OR forest* OR coast* OR ocean* or sea* | Expanders - Apply equivalent subjects  Search modes - Boolean/Phrase | Interface - EBSCOhost Research Databases  Search Screen - Basic Search  Database - MEDLINE with Full Text | 1,861,916 |
| S14 | Outdoor* | Expanders - Apply equivalent subjects  Search modes - Boolean/Phrase | Interface - EBSCOhost Research Databases  Search Screen - Basic Search  Database - MEDLINE with Full Text | 32,802 |
| S13 | (blue OR green) AND (health OR nature OR space) | Expanders - Apply equivalent subjects  Search modes - Boolean/Phrase | Interface - EBSCOhost Research Databases  Search Screen - Basic Search  Database - MEDLINE with Full Text | 101,272 |
| S12 | (MH "Ecosystem+") | Expanders - Apply equivalent subjects  Search modes - Boolean/Phrase | Interface - EBSCOhost Research Databases  Search Screen - Basic Search  Database - MEDLINE with Full Text | 288,024 |
| S11 | (MH "Natural Environment+") OR "natur*" | Expanders - Apply equivalent subjects  Search modes - Boolean/Phrase | Interface - EBSCOhost Research Databases  Search Screen - Basic Search  Database - MEDLINE with Full Text | 2,013,215 |
| S10 | S1 OR S2 OR S3 OR S4 OR S5 OR S6 OR S7 OR S8 OR S9 | Expanders - Apply equivalent subjects  Search modes - Boolean/Phrase | Interface - EBSCOhost Research Databases  Search Screen - Basic Search  Database - MEDLINE with Full Text | 732,244 |
| S9 | (MH "Video-Assisted Techniques and Procedures+") | Expanders - Apply equivalent subjects  Search modes - Boolean/Phrase | Interface - EBSCOhost Research Databases  Search Screen - Basic Search  Database - MEDLINE with Full Text | 12 |
| S8 | 360° video* | Expanders - Apply equivalent subjects  Search modes - Boolean/Phrase | Interface - EBSCOhost Research Databases  Search Screen - Basic Search  Database - MEDLINE with Full Text | 135 |
| S7 | internet*base* interven* | Expanders - Apply equivalent subjects  Search modes - Boolean/Phrase | Interface - EBSCOhost Research Databases  Search Screen - Basic Search  Database - MEDLINE with Full Text | 0 |
| S6 | (MH "Webcasts+") OR "Streaming" | Expanders - Apply equivalent subjects  Search modes - Boolean/Phrase | Interface - EBSCOhost Research Databases  Search Screen - Basic Search  Database - MEDLINE with Full Text | 6,592 |
| S5 | Simulat* natur* | Expanders - Apply equivalent subjects  Search modes - Boolean/Phrase | Interface - EBSCOhost Research Databases  Search Screen - Basic Search  Database - MEDLINE with Full Text | 3,933 |
| S4 | (MH "Simulations+") OR "Simulation*" OR (MH "Computer Simulation+") | Expanders - Apply equivalent subjects  Search modes - Boolean/Phrase | Interface - EBSCOhost Research Databases  Search Screen - Basic Search  Database - MEDLINE with Full Text | 645,770 |
| S3 | (Virtual experiment* environment* OR VEE*) | Expanders - Apply equivalent subjects  Search modes - Boolean/Phrase | Interface - EBSCOhost Research Databases  Search Screen - Basic Search  Database - MEDLINE with Full Text | 31,438 |
| S2 | "(Virtual health room* OR VHR)" | Expanders - Apply equivalent subjects  Search modes - Boolean/Phrase | Interface - EBSCOhost Research Databases  Search Screen - Basic Search  Database - MEDLINE with Full Text | 0 |
| S1 | (MH "Virtual Realit*+") OR "Virtual reality" OR "VR" OR (MM "Virtual Reality Exposure Therapy") | Expanders - Apply equivalent subjects  Search modes - Boolean/Phrase | Interface - EBSCOhost Research Databases  Search Screen - Basic Search  Database - MEDLINE with Full Text | 58,464 |

Bottom of Form

**PsychInfo Search Pattern (115 Results):**

| **#** | **Query** | **Limiters/Expanders** | **Last Run Via** | **Results** |
| --- | --- | --- | --- | --- |
| S31 | S10 AND S16 AND S26 AND S30 | Expanders - Apply equivalent subjects  Search modes - Boolean/Phrase | Interface - EBSCOhost Research Databases  Search Screen - Advanced Search  Database - APA PsycInfo | 115 |
| S30 | S27 OR S28 OR S29 | Expanders - Apply equivalent subjects  Search modes - Boolean/Phrase | Interface - EBSCOhost Research Databases  Search Screen - Advanced Search  Database - APA PsycInfo | 533,257 |
| S29 | student AND (college* OR universit* OR higher educat*) | Expanders - Apply equivalent subjects  Search modes - Boolean/Phrase | Interface - EBSCOhost Research Databases  Search Screen - Advanced Search  Database - APA PsycInfo | 522,319 |
| S28 | Student Health Services | Expanders - Apply equivalent subjects  Search modes - Boolean/Phrase | Interface - EBSCOhost Research Databases  Search Screen - Advanced Search  Database - APA PsycInfo | 1,750 |
| S27 | DE "Higher Education" OR DE "Graduate Education" OR DE "Postgraduate Training" OR DE "Undergraduate Education" | Expanders - Apply equivalent subjects  Search modes - Boolean/Phrase | Interface - EBSCOhost Research Databases  Search Screen - Advanced Search  Database - APA PsycInfo | 31,497 |
| S26 | S17 OR S18 OR S19 OR S20 OR S21 OR S22 OR S23 OR S24 OR S25 | Expanders - Apply equivalent subjects  Search modes - Boolean/Phrase | Interface - EBSCOhost Research Databases  Search Screen - Basic Search  Database - APA PsycInfo | 1,281,116 |
| S25 | DE "Stress" OR DE "Academic Stress" OR DE "Environmental Stress" OR DE "Psychological Stress" OR DE "Stress Reactions" OR MM "Stress and Coping Measures" OR MM "Stress Management" | Expanders - Apply equivalent subjects  Search modes - Boolean/Phrase | Interface - EBSCOhost Research Databases  Search Screen - Basic Search  Database - APA PsycInfo | 99,225 |
| S24 | DE "Anxiety" OR DE "Anxiety Disorders" OR anxiet* | Expanders - Apply equivalent subjects  Search modes - Boolean/Phrase | Interface - EBSCOhost Research Databases  Search Screen - Basic Search  Database - APA PsycInfo | 315,011 |
| S23 | MM "Depression (Emotion)" OR depressi* | Expanders - Apply equivalent subjects  Search modes - Boolean/Phrase | Interface - EBSCOhost Research Databases  Search Screen - Basic Search  Database - APA PsycInfo | 413,253 |
| S22 | "(cognit* OR affect*) AND (health* OR disorder*)" | Expanders - Apply equivalent subjects  Search modes - Boolean/Phrase | Interface - EBSCOhost Research Databases  Search Screen - Basic Search  Database - APA PsycInfo | 0 |
| S21 | "mental* AND restor*" | Expanders - Apply equivalent subjects  Search modes - Boolean/Phrase | Interface - EBSCOhost Research Databases  Search Screen - Basic Search  Database - APA PsycInfo | 10 |
| S20 | "mental disorder*" AND (prevent* OR control*) | Expanders - Apply equivalent subjects  Search modes - Boolean/Phrase | Interface - EBSCOhost Research Databases  Search Screen - Advanced Search  Database - APA PsycInfo | 60,745 |
| S19 | mental AND (problem* OR health difficult*) | Expanders - Apply equivalent subjects  Search modes - Boolean/Phrase | Interface - EBSCOhost Research Databases  Search Screen - Advanced Search  Database - APA PsycInfo | 184,654 |
| S18 | Mental Health research | Expanders - Apply equivalent subjects  Search modes - Boolean/Phrase | Interface - EBSCOhost Research Databases  Search Screen - Advanced Search  Database - APA PsycInfo | 25,163 |
| S17 | DE "Mental Health" OR MM "Preventive Mental Health Services" OR "mental health" | Expanders - Apply equivalent subjects  Search modes - Boolean/Phrase | Interface - EBSCOhost Research Databases  Search Screen - Advanced Search  Database - APA PsycInfo | 733,047 |
| S16 | S11 OR S12 OR S13 OR S14 OR S15 | Expanders - Apply equivalent subjects  Search modes - Boolean/Phrase | Interface - EBSCOhost Research Databases  Search Screen - Advanced Search  Database - APA PsycInfo | 647,741 |
| S15 | landscape* OR forest* OR coast* OR ocean* or sea* | Expanders - Apply equivalent subjects  Search modes - Boolean/Phrase | Interface - EBSCOhost Research Databases  Search Screen - Advanced Search  Database - APA PsycInfo | 272,180 |
| S14 | Outdoor* | Expanders - Apply equivalent subjects  Search modes - Boolean/Phrase | Interface - EBSCOhost Research Databases  Search Screen - Advanced Search  Database - APA PsycInfo | 7,346 |
| S13 | (blue OR green) AND (health OR nature OR space) | Expanders - Apply equivalent subjects  Search modes - Boolean/Phrase | Interface - EBSCOhost Research Databases  Search Screen - Advanced Search  Database - APA PsycInfo | 16,537 |
| S12 | Ecosystem* | Expanders - Apply equivalent subjects  Search modes - Boolean/Phrase | Interface - EBSCOhost Research Databases  Search Screen - Advanced Search  Database - APA PsycInfo | 17,996 |
| S11 | DE "Nature (Environment)" OR natur* | Expanders - Apply equivalent subjects  Search modes - Boolean/Phrase | Interface - EBSCOhost Research Databases  Search Screen - Advanced Search  Database - APA PsycInfo | 373,657 |
| S10 | S1 OR S2 OR S3 OR S4 OR S5 OR S6 OR S7 OR S8 OR S9 | Expanders - Apply equivalent subjects  Search modes - Boolean/Phrase | Interface - EBSCOhost Research Databases  Search Screen - Advanced Search  Database - APA PsycInfo | 88,706 |
| S9 | Video-Assisted Techniques and Procedures | Expanders - Apply equivalent subjects  Search modes - Boolean/Phrase | Interface - EBSCOhost Research Databases  Search Screen - Advanced Search  Database - APA PsycInfo | 0 |
| S8 | 360° video* | Expanders - Apply equivalent subjects  Search modes - Boolean/Phrase | Interface - EBSCOhost Research Databases  Search Screen - Advanced Search  Database - APA PsycInfo | 77 |
| S7 | internet*base* interven* | Expanders - Apply equivalent subjects  Search modes - Boolean/Phrase | Interface - EBSCOhost Research Databases  Search Screen - Advanced Search  Database - APA PsycInfo | 3 |
| S6 | Webcast* OR streaming | Expanders - Apply equivalent subjects  Search modes - Boolean/Phrase | Interface - EBSCOhost Research Databases  Search Screen - Advanced Search  Database - APA PsycInfo | 1,456 |
| S5 | Simulat* natur* | Expanders - Apply equivalent subjects  Search modes - Boolean/Phrase | Interface - EBSCOhost Research Databases  Search Screen - Advanced Search  Database - APA PsycInfo | 538 |
| S4 | DE "Simulation" OR DE "Computer Simulation" OR DE "Simulation Games" OR simulation* | Expanders - Apply equivalent subjects  Search modes - Boolean/Phrase | Interface - EBSCOhost Research Databases  Search Screen - Advanced Search  Database - APA PsycInfo | 68,561 |
| S3 | (Virtual experiment* environment* OR VEE*) | Expanders - Apply equivalent subjects  Search modes - Boolean/Phrase | Interface - EBSCOhost Research Databases  Search Screen - Advanced Search  Database - APA PsycInfo | 5,388 |
| S2 | (Virtual health room* OR VHR) | Expanders - Apply equivalent subjects  Search modes - Boolean/Phrase | Interface - EBSCOhost Research Databases  Search Screen - Advanced Search  Database - APA PsycInfo | 18 |
| S1 | DE "Virtual Reality" OR MM "Virtual Reality Exposure Therapy" OR MM "Virtual Environment" OR "Virtual Realit*" OR "VR" | Expanders - Apply equivalent subjects  Search modes - Boolean/Phrase | Interface - EBSCOhost Research Databases  Search Screen - Advanced Search  Database - APA PsycInfo | 15,126 |

Bottom of Form

# **Table 1: Title, aim, hypothesises, country, design, experimental conditions and allocation**

| **Author** | **Title** | **Aim** | **Hypothesises** | **Country & sample** | **Excluded in current/history mental illness (Y/N)** | **Design** | **Experimental conditions** | **Allocation** |
| --- | --- | --- | --- | --- | --- | --- | --- | --- |
| Alyan 2021 | The Influence of Virtual Forest Walk on Physiological and Psychological Responses | To quantify participants’ moods after viewing different forest environments used to reduce psychological stress. | - | Malaysia,  n=20,  10 (50%) females,  Average age 21.8 years | Y | Between-groups design, measuring independent variables pre- and post- intervention. | Two experimental conditions. Two computer-generated VR Forest environments:  1) dreamlike vs.  2) realistic | Participants randomly assigned to experience one of two conditions. |
| Browning 2020 | Can Simulated Nature Support Mental Health? Comparing Short, Single-Doses of 360-Degree Nature Videos in Virtual Reality with the Outdoors | To determine whether a single dose of 360-degree nature video exposure yields similar benefits as a single dose of physical nature exposure. More precisely, we aim to test where along a spectrum from no nature exposure to extensive nature exposure the psycho-physiological benefits of virtual exposure fall. | - | USA,  n=98  39 (40%) females,  Mean age 20 (SD=1.12) | Y | Between-groups design, measuring independent variables pre- and post- intervention. | Three experimental conditions: 1) a real outdoor forest setting;  2) a 360-degree video of that same forest; or  3) an indoor setting with no visual or auditory access to nature. | Participants were randomly assigned to one of three conditions. |
| Browning 2023 | Daily exposure to virtual nature reduces symptoms of anxiety in college students | To test the impacts of daily exposure over at least a three-week period on college students’ symptoms of anxiety, depression, and rumination. Our primary objective was to evaluate the impacts of daily virtual nature exposure on symptoms relative to no exposure. | - | USA,  n=40,  30 (75%) females,  18 to 22 years (Mean = 19.3, SD = 1.2). | Y | Between-groups design, measuring independent variables pre- and post- intervention. | Two experimental conditions:  1) video VR nature (day 1 Aspen forest, day 2 beaches day 3 forests, day 4 forests with water, day 5 rain forest, day 6 waters vs  2) no intervention | Participants randomly assigned to experience one of two conditions. |
| Chan 2023 | Nature in virtual reality improves mood and reduces stress: evidence from young adults and senior citizens | To investigate if virtual natural environments can promote mental health by improving mood (affect) and reducing stress. | The nature condition:  H1) increases positive affect,  H2) decreases negative affect compared to the urban condition.  H3) the relationship between nature exposure and positive affect will be mediated by nature connectedness,  H4) the relationship between nature exposure and negative affect will be mediated by nature connectedness,  H5) the nature condition would be associated with a lower level of stress compared to the urban condition (H5). | Singapore,  n=12  females 6, age mean 22.6 SD=1.3 | N | Within-subject design where each participant experienced the nature and urban scene on two separate days respectively. | Two experimental conditions. Participants experienced the 1) VR forest, and  2) VR urban environments, with one week in-between, and the order was counter- balanced. | NA |
| Chen 2023 | Investigating the Efficacy of Virtual Reality Forest Meditation as  an Intervention for Test Anxiety among College Students | To verify the  effectiveness of virtual reality forest meditation as an intervention  for test anxiety among college students and to investigate the impact  of three factors: namely forest style, meditation state, and  pranayama guidance, on the intervention effect. | - | China  n=30  female = 30, male = 30 | Y | Within-subject three-factor controlled experiment | Eight experimental forest scenarios consisting of combinations of:   - Real/ non-real style - Sitting / wondering meditation - With / without pranayama guidance | No |
| Gao 2019 | Exploring Psychophysiological Restoration and Individual Preference in the Different Environments Based on Virtual Reality | To investigate  -the difference in the restorative state before and after visual stimulation by using VR devices  -the effects of the different types of environments on people’s physiological and psychological restoration  -the types of environments that people prefer, and how preferences relate to restorative effects of environments | - | China, n=120 females 62  Age mean 20.7  SD 2.13 | Y | Between groups design, measuring variables pre and post intervention | Six VR experimental conditions: Open green space, Partly open green space, Partly closed green space, Closed green space, Blue space, Grey space | Random assignment to one type of environment, with each group containing 20 participants |
| Hsieh 2023 | The effect of water sound level in virtual reality: A study of restorative benefits in young adults through immersive natural environments | This study aimed to address the lack of clarity in the literature regarding the impact of water sounds on VR experiences. | - | Taiwan,  n=45,  29 (64%) females,  19 to 23 years of age | Y | Between-groups design measuring independent variables pre- and post- intervention. | Three experimental conditions:  1) VR forest no waterfall sound (control),  2) VR forest low decibel group,  3) VR forest high decibel group | Participants randomly assigned to experience one condition |
| Jo 2021 | The Effect of Forest Video Using Virtual Reality on the Stress Reduction of University Students Focused on C University in Korea | To investigate the effect of watching forest videos using virtual reality (VR), a modern technology, on the stress reduction of college students and to suggest a stress management plan for college students. | - | South Korea,  n=60,  16 (27%) females,  age not reported | N | Between-groups design measuring independent variables pre- and post- intervention. | Three experimental conditions:  1) 2D (flat screen) forest videos  2) VR forest  3) Group does not watch anything (control) | Groups randomly created to experience one condition |
| Leger 2022 | Simulating the Benefits of Nature Exposure on Cognitive Performance in Virtual Reality: A Window into Possibilities for Education and Cognitive Health | To compare effect of an outdoor nature walk to those of a virtual nature walk | Participating university students would perform as well on memory and executive tests when they experience a virtual reality nature walk, compared to an actual forest nature walk | Canada,  n=81,  46 (57%) females,  20 to 24 years of age | Y | Within-subject design where each subject experienced the VR nature walk and actual forest walk | Two experimental conditions:  1) VR forest walk,  2) actual forest nature walk | NA |
| Li 2020 | Effects of brightness levels on stress recovery when viewing a virtual reality forest with simulated natural light | (1) To investigate whether watching virtual reality forest has stress and anxiety recovery effect, (2) to compare the differences in participants’ psychological responses and psychological effects for different brightness scenes in the virtual reality forest. | - | China,  n=120,  93 (78%) females,  17 to 28 years (M = 19.79, SD = 1.90) | N | Between-groups design measuring independent variables pre- and post- intervention. | Six VR forest experimental conditions with different brightness levels | Randomly assigned to one of six conditions |
| Li 2021 | Effect of a Virtual Reality-Based Restorative Environment on the Emotional and Cognitive Recovery of Individuals with Mild-to-Moderate Anxiety and Depression | To explore the intervention effect of the presence of a VR restorative environment on individuals with mild-to-moderate anxiety and depression from the perspective of emotion and cognition | -There will be differences in subjective restoration and the sense of presence of different  VR restorative scenes. The subjective restoration of VR restorative scenes will be  higher than that of VR urban scene. The presence of VR restorative environment with  interaction will be better than that in other intervention groups.  -Different VR restorative environment experiences will have different healing effects  on the degree of change in individual emotions and self-efficacy for individuals with  mild-to-moderate anxiety and depression.  -VR restorative scenes (Env2~Env5) will contribute to improve positive emotions,  reduce negative emotions, and improve self-efficacy of individuals with mild-to moderate  anxiety and depression. The VR urban scene (Env1) will have the opposite  effect to VR restorative scenes. Different VR restorative environment experiences will  have different healing effects on the directions of change in individual emotions and  self-efficacy.  -The recovery impact of the VR rehabilitative environment on people was probably  realized through the presence of VR scenes.  -The differences of presence will also be reflected in EMG: compared to the baseline,  indicators of physical participation (contraction of the brachioradialis muscle of the  participants’ arm) in the VR scene experience will improve.  -VR restorative scenes will be conductive to the cognitive recovery of individuals with  mild-to-moderate anxiety and depression, which will be reflected in EEG indicators:  prefrontal alertness and engagement will be increased, and the calming signal index  will be decreased in the VR restorative environment experience. | China, n=189, females 113, average age 20.26 years SD 2.58 | N | Between groups design measuring variables pre and post intervention | Study 2:  Four VR restorative garden groups: i) a VR restorative environment visual experiencing group  ii) a VR restorative environment interactive experiencing group  iii) a VR restorative environment with fishing interaction group  iv) and a VR  restorative environment with watering interaction group  and the control group: a VR urban environment | Random assignment to one of the experimental groups |
| Manchon 2023 | Effects of Short-Term Exposure to Interactive  vs. Non-Interactive Virtual Nature on Cognitive  Performance and Mental Health in College  Students | to examine the effects of a 6-minute exposure to nature virtually in a non-interactive and interactive way  on cognitive performance (short-term memory and executive  functions – visual attention/task switching) and the state  of well-being (levels of stress, happiness, sadness, calmness,  tenseness, and energy) | we hypothesize that  short-term exposure to virtual nature environments will  result in greater improvements in cognitive function, stress  reduction, and perceived restoration than not being exposed  to nature (control group). We also hypothesize that the  exposure to interactive virtual environments will be more  beneficial compared to non-interactive virtual nature environments  among participants. This hypothesis proposes that,  because interactive virtual nature environments allow for  active engagement and exploration, they may have a potentially  greater impact on cognitive and emotional well-being  than non-interactive virtual nature environments, which  only offer passive observation. Additionally, since it could be expected that those with a  greater sense of connectedness to nature could have greater  benefits from exposure to natural environments (Leung  et al., 2022; Teixeira et al., 2023), we checked the differences  in the size of the effects of VR nature exposure according to  the type of exposure while controlling for the levels of connectedness  to nature. The size of the effects (calculated difference  between the results before and after exposure) was  expected to be the largest for the group interactively exposed  to VR nature and the lowest for the group not exposed to  any natural environment. | Croatia  N=64 undergrad psychology students  59 women, 5 men, between 18-25 years | N | Between groups design | 1. Control = exposed to a white wall  2. VR nature interactive through a specific VR game called Nature Treks  VR, which allows the player to interact in different natural  environments with their own wildlife and vegetation.  3. VR nature non-interactive 360 VR video of different natural environments | divided into  3 groups of similar size that differed by the type of exposure*.* |
| Mostajeran 2021 | Effects of exposure to immersive videos and photo slideshows of forest and urban environments. | To directly compare conventional photo presentations and 360◦ video presentation of nature. | The environment and the immersion level, as well as their interaction, have an influence on mood, stress recovery, and cognitive performance. In particular, we expected that the forest environment would produce a more positive effect than the urban environment. In addition, we hypothesized that more immersive presentations (i.e., 360◦ videos) create a higher sense of presence and consequently have greater effects. | Germany,  n=34,  11 (32%) females,  21 to 34 years of age (M=27.26, SD=4.144) | N | Within-subjects design measuring independent variables pre- and post- intervention. | A control (a black virtual room with a white screen showing a fixation cross) and four experimental conditions: VR  Environnent  (Forest, Urban)  x Immersion  (Photo  Slideshows,  360-degree  Videos) | NA |
| Mostajeran 2023 | Effects of exposure to immersive computer generated virtual nature and control environments on affect and cognition. | Compare effects of exposure to computer-generated nature environment vs. video of real nature. | In comparison to the video of real nature exposure to computer-generated virtual nature results in:  H1) higher cognitive performance,  H2) higher perceived restorativeness,  H3) higher positive affect,  H4) lower negative affect,  H5) lower stress,  H6) higher sense of presence, and  H7) lower simulator sickness. | Germany, n=27,  12 (44%) females,  21 to 59 years of age (M = 28.15, SD = 8.27) | N | A paired comparison of the post-test measures of the two virtual environments (not the improvements of the measures compared to the baseline). | Two experimental conditions: Computer-generated forest environment vs. the control group - abstract shape representation of forest environment (cylinders, cubes and cuboids) | NA |
| O’Meara 2020 | Virtual Reality Nature Exposure and Test Anxiety | To investigate whether one stint of nature exposure through IVR can  improve the experience of the test and test performance in individuals with test anxiety. | Firstly, that high test anxiety participants’ self-reported negative  affect would be lower following exposure to a green environment via virtual reality and that this change would not be observed by participants who are exposed to an urban environment via virtual  reality.  Second, to test whether positive affect was increased by virtual exploration of a natural scene. | Ireland,  N=40 divided into High Anxiety group (4 males, 16 females) and Low Anxiety group (6 males, 14 females) | Y | Between and within-participants design  (2 (high vs. low anxiety) x 2 (urban vs. nature virtual reality (VR)) x 2 (pre vs. post VR intervention). | 4 experimental groups: 2 (high vs. low anxiety) x 2 (VR nature (forest)  vs. VR urban) | Participants were  assigned to one of the two groups based on their scorings on the test anxiety questionnaire.  Within each group, participants were randomly assigned to either an urban or nature virtual reality  (VR) intervention condition. |
| Reece 2022 | Exposure to Green, Blue and Historic Environments and Mental Well-Being: A Comparison between Virtual Reality Head-Mounted Display and Flat Screen Exposure | To assess whether exposure to a potentially restorative environment (green, blue, and historic), presented either through VR or Flat Screen (FS) FS video, was associated with mental well-being benefits following exposure to a stressor environment (traffic). | _ | England,  n=31,  22 (71%) females,  18 to 57 years (M = 24.32, SD = 8.73) | Y | Between-subjects design – comparing independent variables pre- and post each of the three VR scenarios | Three environment videos (green (open field), blue, (lake) historic) either as 2D flatscreen or VR | Participants randomly assigned to watch the environment videos on flat screen (FS) or using a VR HMD. |
| Weixin 2023 | Research on the Methods of VR Technology to Reduce the Stress of College Students: A Case-study of Virtual Aquarium | To investigate effects on stress of VR aquarium | - | China,  n=10,  3 (30%) females,  Average age 22 years old | N | Repeated measures design | There are 10 different scenes in the virtual aquarium that the researchers set up in advance, which contain different types and numbers of fish. | NA |
| Suseno 2023 | The effect of simulated natural environments in virtual reality and 2D video to reduce stress | To determine the effect of simulated natural environments in virtual reality and 2D video and their differences in reducing stress. | H1) Both simulated natural environments in virtual reality and 2D video can reduce stress,  H2) there is a difference between simulated natural environments in virtual reality and 2D video in reducing stress levels. A simulated natural environment in virtual reality can reduce stress more than a simulated natural environment in a 2D video. | Indonesia  n=53,  24 (45%) females,  The average age of the virtual reality group was 20.7 (SD = 1.72), and the average of the 2D video group was 19.8 (SD = 1.31). | Y | Between-groups design measuring independent variables pre- and post- intervention. | Two experimental conditions:  1) VR Nokia Bay  2) 2D (flat screen) Nokia Bay | Randomly allocated into one of two conditions |
| Theodorou 2023 | Different types of virtual natural environments enhance subjective vitality through restorativeness | To investigate the relationship between distinct virtual natural environments and subjective vitality and the underlying processes that help explain this relationship. | Virtual natural environments, namely a national park, a lacustrine environment, and an arctic environment, enhance restorativeness significantly more than the virtual urban environment, and that, in turn, determines greater subjective vitality. | Italy  n=133  90 (68%) females,  age not reported | N | Between-groups design measuring independent variables pre- and post- intervention. | Four experimental conditions:  1) VR urban environment (control),  2) National Park,  3) Lacustrine environment,  4) Artic environment | Participants were randomly assigned to one of four conditions |
| Valtchanov 2010 | Restorative effects of virtual nature settings | Whether immersion in a virtual computer-generated nature setting could produce restorative effect. | Exposure to natural environments in VR would significantly reduce stress, cognitive fatigue, and negative affect, while increasing positive affect in participants who had recently experienced a stressful event. | Canada,  n=22,  12 (55%) females,  17 to 26 years of age | N | Within-subjects design measuring independent variables pre- and post -intervention | Two experimental conditions: VR forest vs. slideshow (flat screen 2D) of abstract paintings of colours found in nature | NA |
| Villani 2007 | New Technologies for Relaxation: The Role of Presence | To investigate with a nonclinical  sample the role of variables associated with the use of different technologies  together with adequate relaxation therapeutic narratives in stress management. |  | Italy,  N=64 (34 female and 30 male students), aged 21  to 28 years | Not clear | Between subjects design with pre and post treatment | 1. VR Condition: Relaxation  Island supported  by a therapeutic narrative.  2. DVD Condition: tropical videos  supported by the same therapeutic narrative.  3. Audio Condition: An audiotape with the same therapeutic narrative  4. Control group: A group without treatment | Random allocation |
| Villani 2008 | Presence and Relaxation: A Preliminary Controlled Study | To investigate with a non clinical sample  the efficacy of a relaxing narrative through a virtual experience. | H1 There is a significant emotional modification within groups. In particular  we expected an increase of relaxation and a decrease of anxiety.  H2 There is a significant difference between the conditions in terms of  efficacy. In particular, we expected an increase of relaxation and positive emotions  higher in VR condition than in the other groups.  H3 A relationship exists between the sense of presence and increase of  relaxation and positive emotions. This relationship is also related to the medium used. | Italy,  N= 60 participants (30 female and 30 male students), aged 21 to 28 years  (M=24.52, SD=1.75) | Not clear | Within group design with pre and post treatments | 1. VR Condition: Relaxation  Island supported  by a therapeutic narrative.  2. DVD Condition: tropical videos  supported by the same therapeutic narrative.  3. Audio Condition: An audiotape with the same therapeutic narrative | Random allocation |
| You 2023 | Biophilic classroom environments on stress  and cognitive performance: A  randomized crossover study in virtual reality  (VR) | To explore the design of study environment in the Metaverse for students and examine  their health responses to the biophilic elements, we used VR and wearable biomonitoring sensors  to quantify the impacts of both positive and negative factors in built virtual environments  on short-term health (i.e., stress reduction and cognitive function improvement within minutes  or hours after exposure) of university students. Specifically, the study investigated the  immediate physiological and cognitive responses (i.e., during the experiment session) to five  virtual scenes of university classroom (same classroom) with different biophilic elements and  turbidity (i.e., hazy outdoor view) which mimics air pollution visually. | We hypothesized that participants would experience physiological stress reduction and cognitive function improvement after exposures to various biophilic classroom  environments in VR, while the turbid environment would negatively influence participants’  short-term health, or immediate impact after exposure. | China n=30  18-30 years, n=16 female | Y | Randomized crossover  design to achieve the same statistical power with fewer participants i.e., participants  served as their own control group with physiological and cognitive responses being repeatedly  measured. | 5 virtual scenes  including one non-biophilic environment as control  and four biophilic interventions. The biophilic intervention refers to the virtual  scene with biophilic design elements. Intervention 1 is Indoor Green where the classroom  is decorated with green plants and natural materials. Intervention 2, named Outdoor  Green, incorporates outdoor natural view and daylight into indoor space through  windows. In Intervention 3, Turbid Outdoor Green, the outdoor natural view in  Intervention 2: Outdoor Green is blocked with visual turbidity, which is intended to simulate  air pollution. The biophilic elements in Indoor Green and Outdoor Green are combined in  Intervention 4 –Combination | Randomised order of the 5 virtual scenes |
| Zhao 2024 | An Exploration of the Physiological and Psychological  Aspects of Student Anxiety Using a Greenspace Restorative  Environment Based on Virtual Reality: A Controlled  Experiment in Nanjing College | We recruited college students who experienced stress and anxiety and  investigated the effects of restorative environmental components, including the terrain  scene, pavement material, and scene green visual rate, from both physiological and psychological  perspectives by exposing the participants to a greenspace environment created  via VR. We also investigated the restorative effects of greenspace components on students’  anxiety to help develop better services for college students with anxiety. | - | China  n=36  Aged 22-26  1:1 female-to-male ratio | N  participants generally believed that they had mild depression, low self-rated anxiety, and  good health | Separate experiments  Within and between-groups design depending on the experiment | 3 green components:  1. terrain scene,  2. pavement material,  3. scene green visual rate  1. Terrain scene  In Scenario 1 (experimental group), the terrain was altered, whereas all other factors remained constant. Conversely,  Scenario 2 (control) featured a flat field with no terrain modifications.  2. Paving material  The experiment on terrain material compared Scenes 3 and 4, with the only difference  being the paving material. The other factors in the scenes were kept constant. Scenario  3 (experimental group) used natural materials, such as marble, pebbles, granite, wood, and plain soil, while  Scenario 4 (control) used artificial materials, such as cement, asphalt, glass, steel, and concrete.  3. Green visual rate  The obtained green vision rate was divided into four grades  using the quartile method: lower (<37%), low (37%–42%), high (42%–54%), and higher  green vision | Not described |

**Table 2 Subjective psychological outcomes**

| **Author** | **Mood** | **Stress** | **Anxiety** | **Cognition** |
| --- | --- | --- | --- | --- |
| Alyan 2021 | POMS | - | - | - |
| Browning 2020 | PANAS | - | - | - |
| Browning 2023 | Mood and Anxiety Symptom Questionnaire | - | Penn State Worry Questionnaire  Rumination Reflection Questionnaire | - |
| Chan 2023 | PANAS | - | - | - |
| Chen 2023 | - | - | Test Anxiety Scale (TAS) | - |
| Gao 2019 | POMS-SF | - | - | Stroop color task |
| Hsieh 2023 | - | - | State Trait Anxiety Inventory (STAI) | - |
| Jo 2021 | - | - | - | - |
| Leger 2022 | - | - | - | i) Part B of the Trail Making Test to measure executive functioning  ii) Digit Span Test to measure memory functioning |
| Li 2020 | - | - | STAI | - |
| Li 2021 | Self-Rating Depression Scale (SDS) PANAS | - | - | - |
| Manchon 2023 | Visual Analogue Mood Scale | Visual Analogue Scale (VAS) 1-10 |  | i) Executive Function - Trail Making Test (part A&B)  ii) Digital Span Test |
| Mostajeran 2021 | POMS | Short Stress State Questionnaire  Perceived Stress Scale | i) State Trait Anxiety Depression Inventory Scale (STADI) | i) Executive Function - Trail Making Test (part A&B)  ii) Digital Span Test |
| Mostajeran 2023 | Positive and Negative Affect Schedule (PANAS) | Perceived Stress Scale | - | i) Part A & B of the Trail Making Test  ii) Digit Span Test to measure memory functioning |
| O’Meara 2020 | PANAS | - | Test Anxiety Questionnaire | Non verbal reasoning test |
| Reece 2022 | - | University of Wales Institute of Technology Mood Adjective Check List | STAI-S | - |
| Weixin 2023 | - | - | - | - |
| Suseno 2023 | - | - | i) STAI  ii) Autonomic Perception Questionnaire | - |
| Theodorou 2023 | - | - | - | - |
| Valtchanov 2010 | Zuckerman Inventory of Personal Reactions | - | - | Mental arithmetic quizzes |
| Villani 2007 | PANAS | VAS  Coping Orientation to Problems Experienced Questionnaire (COPE) | STAI |  |
| Villani 2008 | PANAS | VAS  Coping Orientation to Problems Experienced Questionnaire (COPE) | STAI |  |
| You 2023 | - | Participants were asked to rate their current stress levels immediately after the exposure to each scene with a range from 1 to 5 (no decimal) where 1 refers to “not stressful” and 5 denotes “extremely stressful.” | - | 1. Measured attentional restoration via the Verbal Backward Digit Span Task (Number test)  2. Guilford’s Alternative Uses test (AU test) for creativity evaluation |
| Zhao 2024 | PANAS | - | - | - |
| Total (n=24) | 14 | 7 | 10 | 8 |

**Table 3: Objective physiological outcomes**

| **Author** | **Cardiovascular** | **SCL** | **EEG** |
| --- | --- | --- | --- |
| Alyan 2021 | HR and blood circulation speed using a blood volume pulse sensor (Thought Technologies, model SA9308M) placed on index finger to assess heart rate beats per minute. | Sensor (Thought Technology Model SA9309M) attached to the index and ring fingers to measure skin conductivity in MicroMho (0-30MΩ) because MicroMho rises in response to an increase in anger-related arousal | - |
| Browning 2020 | - | Shimmer GSR+ sensor nodes attached to the second and fourth finger of participants’ non-dominant hand | - |
| Browning 2023 | - | - | - |
| Chan 2023 | HR & HRV measured by Electrocardiogram (ECG) device from BITalino using electrodes attached under participants right and left collarbones, and lower left ribcage at a sampling rate of 1000 Hz. | - | - |
| Chen 2023 | - | - | EEG the α  wave associated with a relaxed and clear thinking  state, and the β  wave linked to high mental tension.  The experimental  setup utilized Emotiv Epoc+, a portable EEG device manufactured  by an American Neurotechnology Company. This device features  14 wet electrode channels and operates at a sampling rate of 256  samples per second multiplied by 2048 hertz |
| Gao 2019 | - | - | EEG NeuroSky portable brainwave device using electrodes |
| Hsieh 2023 | Heart beat monitor sensor (BeneGear, New Taipei City, Taiwan) | - | - |
| Jo 2021 | HRV measured through light reflection changes in the haemoglobin of fingertip capillaries. Participants wore a clip on the index finger from Ubiomacpa/UBioClip v40. | - | EEG device BIOS-ST (BioBrain Inc.) |
| Leger 2022 | - | - | - |
| Li 2020 | HR and blood circulation speed using Biofeedback System (Thought Technology Ltd) cardiovascular sensor placed on the middle finger. The blood cells reflection was sensed by a cardiovascular sensor emitting infrared waves. | Sensor (Thought Technology Ltd) connected to the participants’ index and middle finders with electrodes. | - |
| Li 2021 | - | - | EEG100C amplifier |
| Manchon 2023 | - | - | - |
| Mostajeran 2021 | HR (method not described) | Galvanic Skin Response sensors on finger of non-dominant hand | - |
| Mostajeran 2023 | - | - | - |
| O’Meara 2020 | - | - | - |
| Reece 2022 | - | - | Non-invasive cap with 32 electrodes and gel |
| Weixin 2023 | HR tool (Runtustic heart rate) downloaded from Apple App store and measured by participant placing finger on rear camera lens and flashlight | - | - |
| Suseno 2023 | HR & BVP using ProComp5 Infiniti biofeedback system (Thought Technology Ltd) using a sensor on middle finger | ProComp5 Infiniti biofeedback system (Thought Technology) using sensor on the index and ring fingers. | - |
| Theodorou 2023 | **-** | **-** |  |
| Valtchanov 2010 | HR (PowerLab Data Acquisition System (AD Instruments, Colorado Springs) using fingertip sensor placed on ring finger in non-dominant hand | PowerLab Data Acquisition System (AD Instruments, Colorado Springs) using two fingertip electrodes placed on the middle fingers of non-dominant hand | - |
| Villani 2007 | Heart rate and amplitude | Not described | - |
| Villani 2008 | Heart rate and amplitude | Not described | - |
| You 2023 | We used four physiological indicators to assess participants’ acute stress reaction (blood pressure  (BP), heart rate (HR), heart rate variability (HRV), which were obtained either by wearable biomonitoring sensors or calculation.  BP: Omron J760 blood pressure monitor  HR& HRV: The electrodermal activity sensor, Shimmer3 GSR+Unit was attached to participants’ left earlobe to collect the Photoplethysmography (PPG) data | Skin conductance level (SCL), which was obtained either by wearable biomonitoring sensors or calculation. Shimmer3 GSR+Unit was worn on two fingers (middle and ring finger) on participants’ left hands to collect the SCL | - |
| Zhao 2024 | An ECG sensor was used to measure the HRV of the subjects to obtain data | A physiological test module skin electrical  sensor, EDA, was used to measure the skin conductance response of the participants | RMSSD was used for a short-term electrocardiogram (ECG) signal analysis and is  a sensitive indicator of parasympathetic nerve function |
| Total (n= 24) | 13 | 10 | 6 |

**Table 4: VR nature components**

| **Author** | **Environment** | **Computer-generated or 360^o^ image of real nature** | **Sound** | **Other sensory stimuli** | **Interaction in the VR** | **Equipment** | **Exposure (mins)** | **Dose** | **Assess immersion/presence?** |
| --- | --- | --- | --- | --- | --- | --- | --- | --- | --- |
| Alyan 2021 | Forest | Computer-generated imagery (CGI) | Birdsong, flowing water | No | Participants burst bubbles with a touchpad | HMD | 5 | 1 | No |
| Browning 2020 | Forest | 360^◦^ image | Birdsong, flowing water, distant sounds of people and vehicles | No | No | HMD | 6 | 1 | No |
| Browning 2023 | i) Forests (Aspen & rainforest) ii) beaches, iii) deserts, iv) waters e.g. lakes, v) grasslands vi) alpine environment  (with no people in videos) | 360^◦^ image | Nature soundscape with traffic noises removed | No | No | HMD | 4 | Daily for 4 weeks | No |
| Chan 2023 | Forest (no people, animals, water) | CGI | Wind (no birdsong or traffic) | No | Control handles to simulate walking along a path | HMD | 4 | 1 | No |
| Chen 2023 | Forest | CGI | No | No | No | HMD | 3 | 1 | No |
| Gao 2019 | Grey space, Blue space, Green space (further subdivided into four different green space types) | 360^◦^ image | No | No | No | VR glasses | 5 | 1 | No |
| Hsieh 2023 | Forest scene (limiting people) | 360^◦^ image | Waterfall sounds (limiting sounds from people & wind) | No | No | HMD | 5 | 1 | No |
| Jo 2021 | Forest | 360^◦^ image | No | No | No | HMD | 5 | Once a week for 4 weeks | No |
| Leger 2022 | Forest | 360^◦^ image | Typical sounds heard during outdoor nature walk in a forest | No | Walk on a treadmill | HMD | 5 | 1 | No |
| Li 2020 | Forest (people, animals, artificial structures, distinct weather (e.g., fog and rain) not present) | CGI | No | No | No | HMD | 6 | 1 | No |
| Li 2021 | Urban and restorative environments (lawn, garden, water, and forest) | CGI | No | No | Forward, back, direction adjustment, and pull the trigger | HMD | 10 | 1 | Yes |
| Manchon 2023 | Different natural environments. | CGI & 360^o^ image | Yes but not described | No | Interaction with vegetation and animals vs. non-interactive | HMD | 6 | 1 | No |
| Mostajeran 2021 | Forest- vegetation only (water, animals & human not present) | 360^◦^ image | No | No | No | HMD | 6 | 1 | Yes |
| Mostajeran 2023 | Forest | CGI | Birdsong & sound of moving wooden cart | No | No – (not interactive but movement via a virtual wooden cart to transport participants from the start to end of the virtual road) | HMD | 10 | 1 | Yes |
| O’Meara 2020 | Forest | 360^◦^ image | Accompanying sound | No | No | HTC Vive headset with headphones | 4 | 1 | No |
| Reece 2022 | i) green environment – open field, clear and sunny ii) blue environment – large lake, vegetation visible, cloudy with some sun. (people removed in both environment) | 360^◦^ image | i) green environment - sound of wind ii) blue environment - sound of water | No | No | HMD | 2 | 1 | Yes |
| Weixin 2023 | Aquarium | CGI | No | No | Participant can act as visitor in virtual aquarium, replacing different types and different numbers of fish | - | - | - | No |
| Suseno 2023 | Nokia Bay (waterfalls, trees, grass, foliage, rocks, sky) | CGI | Water and birdsong | Wind via a fan | Control button to move to locations in the VR environment | HMD | 3min 25 secs | 8 | No |
| Theodorou 2023 | i) national park ii) lacustrine environment iii) artic environment | 360^◦^ image | No | No | No | HMD | 4 | 1 | No |
| Valtchanov 2010 | Forest (flowers, trees, water, rocks) | CGI | Ambient sounds | ‘Forest breeze’ *Wick* air freshener | i) Self-locomotion within the environment via use of a wireless mouse.  ii) A rumble platform configured to vibrate whenever a ‘step’ was taken within the VR, as well as whenever there was impact between the virtual body and an object e.g. bumping into a tree | HMD | 10 | 1 | No |
| Villani 2007 | Island with ocean and waterfall | 360^◦^ image | Therapeutic narrative | - | No | HMD | 3 | 2 | Yes |
| Villani 2008 | Island with ocean and waterfall | 360^◦^ image | Relaxing narrative | - | No | HMD | Not mentioned | 2 | Yes |
| You 2023 | 5 virtual classroom environments with different nature elements | CGI | No | No | No | HMD | 3 | 1 | No |
| Zhao 2024 | Urban park | CGI | No | No | No | HMD | 5 | 1 | No |

**Table 5: Rationale for VR nature and theories**

|  | **Rationale** | | | | | **Theories of nature** | | | | **Constructs measured** |
| --- | --- | --- | --- | --- | --- | --- | --- | --- | --- | --- |
| **Author** | **Mental health** | **Physical health** | **Cognition** | **Access** | **Environment** | **Attention restoration theory** | **Biophilia hypothesis** | **Stress recovery theory** | **Other** |  |
| Alyan 2021 | x | **-** | x | x | **-** | - | - | x | - | - |
| Browning 2020 | x | **-** | x | x | **-** | x | - | x | Scanning for threats theory | Perceptions of beauty in nature  Restorativeness (using perceived restorativeness scale) |
| Browning 2023 | x | - |  | x | - | - | - | - | - | Perceptions of beauty & disgust in nature |
| Chan 2023 | x | - | x | x | - | x | x | x | - | Nature connectedness |
| Chen 2023 | x | - | - | - | - | - | - | - | - | - |
| Gao 2019 | x | x | x | - | x | x | - | x | - | - |
| Hsieh 2023 | x | - | - | x | - | - | - | - | - | - |
| Jo 2021 | x | - |  | x | - | x | - | x | - | - |
| Leger 2022 | - | - | x | - | - | - | - | - | - | - |
| Li 2020 | x | x | - | - | x | - | - | - | - | Perceived environmental features |
| Li 2021 | x | x | - | - | x | x | - | x | - | Restorativeness (using perceived environmental scale) |
| Manchon 2023 | x | - | x | x | - | x | x | x | - | Nature connectedness |
| Mostajeran 2021 | x | - | - | x | x | x | - | - | - | - |
| Mostajeran 2023 | x | x | x | x | - | x | - | - | - | Restorativeness (using perceived restorativeness scale) |
| O’Meara 2020 | x | - | x | x | - | x | - | x | - | Nature connectedness |
| Reece 2022 | x | - | - | x | - | x | - | x | - | - |
| Weixin 2023 | x | x | - | - | - | x | x | x | - | - |
| Suseno 2023 | x | - | - | x | x | - | - | - | - | - |
| Theodorou 2023 | x | - | x | - | - | x | - | x | - | Restorativeness (using perceived restorativeness scale) |
| Valtchanov 2010 | x | - | x | - | - | x | - | x | - | - |
| Villani 2007 | x | - | - | - | - | - | - | - | Bandura’s theory on self-efficacy | - |
| Villani 2008 | x | - | - | - | - | - | - | - | - | - |
| You 2023 | x | **-** | x | - | **-** | x | x | x | - | Feelings of the connection with nature in the virtual classroom scenes (score 1 ~ 10) |
| Zhao 2024 | x | - | x | - | - | x | - | x | - | Restorativeness (using perceived restorativeness scale) |
| Total (n=21) | 23 | 5 | 12 | 12 | 5 | 15 | 4 | 13 | 2 | Restorativeness n=5  Nature connectedness n=4  Perceptions of the environment n=3 |

**Table 6: Quality Assessment Table**

| Study No. | Q1 | Q2 | Q3 | Q4 | Q5 | Q6 | Q7 | Q8 | Q9 | Q10 | Q11 | Q12 | Q13 | Q14 | Q15 | Q16 | Q17 | Q18 | Q19 | Q20 | Q21 | Q22 | Q23 | Q24 | Q25 | Q26 | Q27 | Overall |
| --- | --- | --- | --- | --- | --- | --- | --- | --- | --- | --- | --- | --- | --- | --- | --- | --- | --- | --- | --- | --- | --- | --- | --- | --- | --- | --- | --- | --- |
| 1 | + | + | + | + | ++ | + | + | - | - | + | - | - | - | + | ? | ? | + | + | + | + | + | + | + | ? | + | ? | ? | 18 |
| 2 | + | + | + | + | ++ | + | + | - | - | + | - | - | - | + | ? | + | + | + | - | + | + | ? | + | ? | + | ? | + | 18 |
| 3 | + | + | + | + | ++ | + | + | - | + | + | - | ? | ? | ? | ? | + | + | ? | ? | + | - | - | + | ? | + | ? | ? | 15 |
| 4 | + | + | - | + | - | + | + | + | + | + | ? | ? | ? | - | ? | + | - | ? | + | + | + | + | - | - | - | + | + | 15 |
| 5 | + | + | + | + | + | + | + | - | + | + | ? | - | - | + | ? | + | + | - | - | + | + | + | + | ? | + | + | + | 19 |
| 6 | + | + | + | + | - | + | + | - | + | + | - | - | ? | ? | ? | + | + | + | - | + | + | ? | + | ? | - | + | ? | 15 |
| 7 | + | + | + | + | ++ | + | + | - | + | + | - | - | - | ? | ? | + | + | + | + | + | + | ? | + | ? | - | + | ? | 18 |
| 8 | + | + | + | + | ++ | + | + | - | + | + | ? | ? | - | ? | ? | + | + | + | + | + | + | ? | + | ? | ? | + | ? | 18 |
| 9 | + | + | + | + | ++ | + | + | - | + | + | - | - | - | ? | - | + | + | + | - | + | + | ? | - | - | + | + | ? | 17 |
| 10 | + | + | + | + | ++ | + | + | - | - | + | - | - | - | ? | ? | + | + | + | - | + | + | ? | + | ? | + | + | ? | 17 |
| 11 | + | + | + | + | ++ | + | + | + | - | + | - | - | ? | ? | ? | + | + | + | ? | + | + | ? | + | ? | - | ? | ? | 16 |
| 12 | + | + | + | + | - | + | + | + | + | + | ? | ? | ? | ? | ? | + | + | + | - | + | + | ? | + | ? | - | + | - | 16 |
| 13 | + | + | + | + | ++ | + | + | + | + | + | ? | - | - | ? | ? | + | + | + | + | + | + | ? | - | - | - | + | ? | 18 |
| 14 | + | + | + | + | + | + | + | + | + | + | ? | ? | - | - | ? | + | + | - | + | + | + | ? | - | ? | - | + | + | 17 |
| 15 | + | + | + | + | ++ | + | + | + | + | + | - | - | - | ? | ? | + | + | + | + | + | + | + | + | ? | - | + | ? | 20 |
| 16 | + | + | + | + | - | + | - | - | - | - | + | + | - | - | - | + | ? | - | ? | + | + | ? | - | - | - | ? | ? | 10 |
| 17 | + | + | + | + | - | + | + | - | + | + | - | - | - | ? | - | + | + | + | - | + | + | ? | + | ? | - | + | ? | 15 |
| 18 | + | + | + | + | ++ | + | + | + | + | + | - | - | - | ? | + | + | + | + | + | + | ? | + | ? | ? | + | + | + | 21 |
| 19 | + | + | + | + | - | + | + | - | - | + | ? | ? | - | ? | ? | + | + | + | ? | + | + | ? | + | ? | - | ? | ? | 13 |
| 20 | + | + | + | + | ++ | + | + | - | + | ? | ? | - | ? | ? | + | - | + | + | + | + | + | - | + | ? | - | ? | - | 15 |
| 21 | + | + | + | + | - | + | + | - | + | + | ? | ? | - | ? | - | + | + | + | + | + | + | ? | + | ? | - | + | ? | 16 |
| 22 | + | + | + | + | - | + | + | + | - | + | - | - | - | + | ? | + | + | + | ? | + | + | ? | + | ? | + | + | + | 18 |
| 23 | + | + | - | + | - | + | - | - | ? | - | ? | ? | ? | ? | ? | + | + | + | ? | + | + | ? | + | ? | ? | ? | + | 11 |
| 24 | + | + | - | + | - | + | + | + | - | - | - | - | - | ? | ? | + | + | + | ? | + | + | ? | + | ? | ? | ? | + | 13 |

Yes = Green box = + , No = Red box = - , Unable to determine = Yellow box = ?

**Summary:**

**Excellent (28-26) - None**

**Good (25-20) – 2 studies**

**Fair (19-15) – 18 studies**

**Poor (14 or below) – 4 studies**

1. Alyan et al., 2021
2. Browning et al., 2020
3. Browning et al., 2023
4. Chan et al., 2023
5. Chen et al., 2023
6. Ghao et al., 2019
7. Hsieh et al., 2023
8. Jo et al., 2021
9. Leger & Mekali, 2022
10. Li et al., 2020
11. Li et al., 2021
12. Manchon et al., 2024
13. Mostajeran et al., 2021
14. Mostajeran et al., 2023
15. Reece et al., 2022
16. Ren et al., 2021
17. Suseno & Hastjarjo, 2023
18. Theodorou et al., 2023
19. Valchanov et al., 2010
20. You et al., 2023
21. Zhao et al., 2024
22. O’Meara et al., 2020
23. Villani et al., 2007
24. Villani et al., 2008

**Table 7: Effect on mental health outcomes**

|  |  | **Self-reported** | | | | **Physiological** | | |
| --- | --- | --- | --- | --- | --- | --- | --- | --- |
| **Author** | **Experimental conditions** | **Mood** | **Anxiety** | **Stress** | **Cognition** | **Cardiovascular** | **Skin conductivity** | **Brain** |
| Alyan 2021 | Two experimental conditions. Two computer-generated VR nature environments:  1) dreamlike vs.  2) realistic | CGI of nature improved mood and stress; realistic compared to dreamlike CGI of nature was significantly more effective in improving mood |  |  |  | CGI of nature reduced stress, realistic rather than dreamlike CGI nature was more significantly effective in reducing stress levels measured by HR | CGI of nature improved mood and stress; realistic rather than dreamlike CGI of nature was more significantly effective in reducing stress levels measured by SCL |  |
| Browning 2020 | Three experimental conditions: 1) a real outdoor forest setting;  2) a 360-degree video of that same forest; or  3) an indoor setting with no visual or auditory access to nature. | 360^◦^ nature did not improve positive affect whereas real outdoor nature and indoor setting with no stimuli did; all three experimental conditions reduced negative affect and there were no significant differences in reductions of negative affect between conditions |  |  |  |  | 360^◦^ nature reduced stress measured by SCL; there were no significant differences in stress levels between the following three experimental conditions: real outdoor nature, 360^◦^ nature and being indoors with no nature stimuli measured by SCL |  |
| Browning 2023 | Two experimental conditions:  1) video VR nature vs  2) no intervention | 360^◦^ nature compared to no intervention significantly reduced worry but there were no significant differences between conditions for other mental health constructs including panic, rumination, and depressive symptoms | 360^◦^ nature compared to no intervention significantly reduced worry but there were no significant differences between conditions for other mental health constructs including panic, rumination, and depressive symptoms |  |  |  |  |  |
| Chan 2023 | Two experimental conditions. Participants experienced the 1) VR nature, and  2) VR urban environments, with one week in-between, and the order was counter- balanced. | CGI nature compared to CGI urban environment significantly decreased negative affect but there were no significant differences between conditions in positive affect |  |  |  | CGI nature reduced stress but compared to urban environment, VR nature did not significantly lower levels of stress measured by cardiovascular activity (ECG and HR) |  |  |
| Chen 2023 | Eight experimental scenarios consisting of   - Real/ non-real style - Sitting / wondering meditation - With / without pranayama guidance |  | Compared to baseline, test anxiety levels significantly decreased after subjects were exposed to VR forest meditation |  |  |  |  | VR forest meditation was effective in alleviating test anxiety. In addition, the intervention effect of wandering meditation was superior to that of sitting meditation and the intervention effect of guided pranayama (R = 0.53 1.35) is superior to that of interventions without pranayama guidance |
| Gao 2019 | Six experimental conditions: Open green space, Partly open green space, Partly closed green space, Closed green space, Blue space, Grey space | The six VR scenes in the experiment led to a significant difference in negative mood restoration, with partly open green space having the greatest reduction in negative mood. No significant difference was noted in positive mood restoration |  |  | VR nature restored attentional fatigue but there were no significant differences noted in terms of attentional fatigue improvement across the six different scenes |  |  | No significant differences were noted in terms of improvement of physiological stress across the six different scenes |
| Hsieh 2023 | Three experimental conditions:  1) VR nature no sound (control),  2) VR nature low decibel group,  3) VR nature high decibel group |  | Both low and high-decibel sound VR nature lowered anxiety. There were no significant differences between 360^◦^ nature with no sound vs. with high decibel water sound vs. with low decibel water sound for anxiety; there were significant differences in emotional arousal with low sound levels calming emotions better than high decibel or no sound conditions |  |  | For most cardiovascular outcomes, there were no significant differences between the three experimental conditions: 360^◦^ nature with no sound *vs*. with high decibel water sound *vs*. with low decibel water sound; however, there were significant differences in LF/HF and LF ratios among the three conditions, with low and high decibel conditions activating the parasympathetic nervous system |  |  |
| Jo 2021 | Three experimental conditions:  1) 2D (flat screen) nature videos  2) VR nature  3) Group does not watch anything (control) |  |  |  |  | The 360^◦^ forest group had a significantly more positive effect on HR parameters than the control group (no intervention), suggesting that the 360^◦^ forest reduced stress |  | A study that measured brain activity to assess participants’ attention, found significant within group pre-post test differences, suggesting that participants were in a higher immersive and concentration state after experiencing 360^◦^ forest |
| Leger 2022 | Two experimental conditions:  1) VR nature walk,  2) actual forest nature walk |  |  |  | Both 360^◦^ nature walk and real nature walk improved cognition but there was not a significant difference between these two conditions |  |  |  |
| Li 2020 | Six experimental conditions with different brightness levels |  | CGI forest environments with day light scenes (i.e. light but not lighter and lightest) significantly reduced anxiety compared to the darkest (i.e. darkest but not darker and dark) night scenes |  |  | CGI forest environments with daylight scenes showed a significant increase in BVP than dark night scenes | CGI forest environments with daylight scenes showed a significant decrease on SCL than dark night scenes |  |
| Li 2021 | Five experimental groups: VR urban environment visual experiencing, VR restorative environment visual experiencing, VR restorative environment interactive experiencing, VR restorative environment with fishing interaction, and VR restorative environment with watering interaction | The five VR environment scenes all led to a significant reduction in negative emotions |  |  |  |  |  | Prefrontal alertness and engagement were significantly increased and the calming signal index was decreased in the VR restorative environment experience, indicating that the prefrontal lobe was activated and in an excited state compared to the baseline state. Differences in effects were noted on the prefrontal lobe across the five scenes, although no mechanism was established |
| Manchon 2023 | 1. Control = exposed to a white wall  2. VR nature interactive  3. VR nature non-interactive | The level of happiness decreased, and sadness increased in the control group in comparison to the two groups exposed to VR nature, where happiness increased and sadness decreased |  | The change or decrease of levels of stress and tenseness was more pronounced in the group exposed to nature interactively than the control group and group exposed to nature non-interactively | No statistically significant pre-post change was found and no differences were found between the control and interactive and non-interactive VR nature conditions |  |  |  |
| Mostajeran 2021 | Four experimental conditions: i) VR urban  ii) slideshow (2D flat screen) urban  iii) VR Forest  iv) slideshow (2D, flat screen) Forest  and v) one control environment: a silent black virtual room with a white screen in the middle showing a fixation cross. | Type of environment (forest *vs* urban) was significantly associated with mood; the forest environment compared to urban environment resulted in a reduction of mood disturbance. In particular, the feeling of fatigue was increased after exposure to the urban environment regardless of their type of presentation (360^◦^ *vs* 2D photo slideshows) and was reduced by exposure to the 360^◦^ videos of forest | No significant main or interaction effects were found for anxiety for the environment (forest vs. urban) and immersive (360^◦^ vs flat screen) four experimental conditions | No significant main or interaction effects were found for stress for the environment (forest vs. urban) and immersive (360^◦^ vs flat screen) four experimental conditions | The forest environment (in 2D photo slideshows and in 360^◦^) significantly improved cognition compared to the urban environment; there was no significant difference on the effect on cognition between forest environment in 2D photo slideshows and 360^◦^ video | No significant main or interaction effect could be observed for the HR difference scores. That is, all four conditions (forest vs. urban) and  360^◦^ and flat screen decreased HR with no significant difference | Galvanic skin response difference scores were significantly larger for the 2D photo slideshow conditions compared to the 360^◦^ conditions. Urban and forest photo slideshows caused significantly larger difference scores compared to 360^◦^ of the urban environment, which suggests that photo slideshows were more effective in lowering arousal levels compared to 360◦ videos |  |
| Mostajeran 2023 | Two experimental conditions: Computer-generated nature environment vs. abstract objects (cylinders, cubes and cuboids) | CGI nature compared to CGI abstract objects environment (cylinders, cubes and cuboids) was significantly more effective in improving positive affect but there were no significant differences in reductions of negative affect between these two conditions. |  | CGI nature compared to VR abstract objects environment (cylinders, cubes and cuboids) was significantly more effective in lowering stress. | CGI nature compared to VR abstract objects environment (cylinders, cubes and cuboids) were associated with significantly higher cognitive performance |  |  |  |
| O’Meara 2020 | Experimental (high anxiety) group and a control (low anxiety) group | No significant effect on postive affect  VR nature led to a significant decrease in negative affect scores for the high anxiety group only | No significant effect on anxiety |  | No significant effect on test scores |  |  |  |
| Reece 2022 | Two experimental conditions:  1) viewing on flat screen (2D), or  2) viewing on VR,  three environment videos (green, blue historic) |  | All VR nature scenes were associated with a reduction in anxiety. There were no significant differences in changes pre-post exposure for anxiety between the three 360^◦^ environmental conditions (green, blue and historic) except for the historic environment which was significantly associated with a smaller reduction of state anxiety compared with the blue environment; there was no significant difference for changes in anxiety between 360^◦^ exposure and flat-screen exposure  Compared to the traffic video, anxiety levels were noted to be lower in the Green, Blue, and Historic conditions | There were no significant differences on stress reduction between the three 360 degree environmental conditions. No differences between flat-screen and VR exposure |  |  |  | The relaxation effect measured by brain activity was contradictory, with some frequency bands showing significant differences between the experimental environment conditions (green vs blue vs historic) while other frequency bands did not. The study also found that brain activity was generally higher for 360^◦^ than flat-screen, and there was evidence of a significant difference across three of the four frequency bands, leading the authors to suggest that this may reflect greater relaxation effects |
| Weixin 2023 | There are 10 different scenes in the virtual aquarium that the researchers set up in advance, which contain different types and numbers of fish. |  |  |  |  | The type and quantity of fish in a virtual aquarium affected people’s feelings and heart rhythm. When people sat in front of a computer program and watched a CGI aquarium scene composed of various fishes, they became more active and calmer, which was evidenced by the decrease in HR; however, the study did not test for differences between conditions |  |  |
| Suseno 2023 | Two experimental conditions:  1) VR Nokia Bay  2) 2D (flat screen) Nokia Bay |  | CGI natural environments and 2D video significantly reduced anxiety measured by STAI & HR; however, there was no difference between the two groups regarding stress reduction |  |  | CGI natural environments and 2D video significantly reduced anxiety measured by STAI & HR; however, there was no difference between the two groups regarding stress reduction | CGI natural environments in VR and 2D video significantly reduced stress measured by SCL; however, there was no difference between the two groups regarding stress reduction |  |
| Theodorou 2023 | Four experimental conditions:  1) VR urban environment (control),  2) National Park,  3) Lacustrine environment,  4) Artic environment |  |  |  |  |  |  |  |
| Valtchanov 2010 | Two experimental conditions: VR nature setting vs. slideshow (flat screen 2D) of abstract paintings of colours found in nature | CGI nature compared to a slideshow (flat screen 2D) of abstract paintings significantly increased positive affect but there were no significant differences in reductions of negative affect between these two conditions |  |  | No statistically significant pre-post change was found and no significant differences for cognition between CGI nature and slideshow (flat screen 2D) of abstract paintings | HR reduced but there was no significant difference in effect on HR between a slideshow (flat screen 2D) of abstract paintings and CGI nature | A slideshow (flat screen 2D) of abstract paintings had no significant effect on SCL whereas there was a significant decrease in SCL after exposure to CGI nature |  |
| Villani 2007 | 4 experimental groups:  1) VR group  2) DVD group  3) Audio group  4) Control group | Not reported | VR nature led to a significant within-group decrease in anxiety. No significant differences were found between the four groups. | VR led to an improvement in ‘relaxing’ state.  No significant differences were found between the four groups. |  | VR did not lead to a significant decrease in HR.  No significant differences were found between the four groups. | VR did not lead to a significant increase in skin response.  No significant differences were found between the four groups. |  |
| Villani 2008 | 4 experimental groups:  1) VR group  2) DVD group  3) Audio group  4) Control group | Not reported | VR nature led to a significant within-group decrease in anxiety. No significant differences were found between the four groups. | VR nature led to a significant within-group increase in ‘relaxing’ state. No significant differences were found between the four groups. |  | VR did not lead to a significant decrease in HR. | VR did not lead to a significant increase in skin response. |  |
| You 2023 | 5 virtual scenes  including one non-biophilic environment as control  and four biophilic interventions. The biophilic intervention refers to the virtual  scene with biophilic design elements. Intervention 1 is Indoor Green where the classroom  is decorated with green plants and natural materials. Intervention 2, named Outdoor  Green, incorporates outdoor natural view and daylight into indoor space through  windows. In Intervention 3, Turbid Outdoor Green, the outdoor natural view in  Intervention 2: Outdoor Green is blocked with visual turbidity, which is intended to simulate  air pollution. The biophilic elements in Indoor Green and Outdoor Green are combined in  Intervention 4 –Combination |  |  | There were statistically significant differences in the self-reported stress levels – all the four biophilic interventions were related to a decreasing self-reported stress level compared to the control non-biophilic scene | No statistically significant pre-post change was found for attention and creativity in all the five different VR classroom scenes with and without biophilic nature components | No statistically significant pre-post change was found for all the physiological indicators (BP, HR, HRV) in all the five different VR classroom scenes with and without biophilic nature components | No statistically significant pre-post change was found for SCL in all the five different VR classroom scenes with and without biophilic nature components |  |
| Zhao 2024 | 3 green components:  1. terrain scene,  2. pavement material,  3. scene green visual rate  1. Terrain scene  In Scenario 1 (experimental group), the terrain was altered, whereas all other factors remained constant. Conversely,  Scenario 2 (control) featured a flat field with no terrain modifications.  2. Paving material  The experiment on terrain material compared Scenes 3 and 4, with the only difference  being the paving material. The other factors in the scenes were kept constant. Scenario  3 (experimental group) used natural materials, such as marble, pebbles, granite, wood, and plain soil, while  Scenario 4 (control) used artificial materials, such as cement, asphalt, glass, steel, and concrete.  3. Green visual rate  The obtained green vision rate was divided into four grades  using the quartile method: lower (<37%), low (37%–42%), high (42%–54%), and higher  green vision | There were no significant differences between participants in the group that had different terrain scenes vs. no change in terrain scene on positive or negative affect and there were no significant differences in the group that had natural material pavement vs. those that had artificial materials. There were no significant differences in positive emotion but there were significant differences in negative emotion between the different green vision levels with higher green vision associated with improving negative emotions |  |  |  | There were no significant differences between participants in the group that had different terrain scenes vs. no change in terrain scene on HRV. There were no significant differences in the group that had natural material pavement vs. those that had artificial materials, and there were no significant differences between the different green vision levels | There were no significant differences between participants in the group that had different terrain scenes vs. no change in terrain scene on skin electrical activity. The natural material pavement scene had a better effect on the improvement of skin electrical activity, and there were no significant differences between the different green vision levels |  |

**Table 8: Effect on presence, restorativeness, nature connectedness, perceptions of environment**

| **Author** | **Experimental conditions** | **Presence** | **Restorativeness** | **Nature connectedness** | **Perceptions of environment** |
| --- | --- | --- | --- | --- | --- |
| Browning 2020 | Three experimental conditions: 1) a real outdoor forest setting;  2) a 360-degree video of that same forest; or  3) an indoor setting with no visual or auditory access to nature. |  | Both real outdoor nature and 360^◦^ nature environments significantly improved restorativeness compared to an indoor environment with no nature stimuli |  | Higher perceptions of beauty in nature moderated the relationship between 360^◦^ nature and positive affect |
| Browning 2023 | Two experimental conditions:  1) video VR nature vs  2) no intervention |  |  |  | Higher perceptions of beauty in nature moderated the relationship between 360^◦^ nature and worry |
| Chan 2023 | Two experimental conditions. Participants experienced the 1) VR nature, and  2) VR urban environments, with one week in-between, and the order was counter- balanced. |  |  | Nature connectedness did not mediate the relationship between CGI nature and positive affect but did between VR nature and negative affect |  |
| Li 2020 | Six experimental conditions with different brightness levels |  |  |  | Compared with the night-time CGI forest environment, perceived environmental feature scores significantly increased in the CGI forest daytime environment, suggesting that daylight promotes perceived safety, visibility, and accessibility |
| Li 2021 | Five experimental groups: VR urban environment visual experiencing, VR restorative environment visual experiencing, VR restorative environment interactive experiencing, VR restorative environment with fishing interaction, and VR restorative environment with watering interaction | One study found from EMG findings that scene experience led to greater arm muscle contraction and thus, greater involvement of the body, indicating different sense of presence in the different scenes. The study also found that sense of presence mediated the effect of restorativeness on positive and negative affect | No significant differences were noted in terms of environmental restoration across the VR scenes, but the VR urban visual scene group scored the highest |  |  |
| Manchon 2023 | 1. Control = exposed to a white wall  2. VR nature interactive  3. VR nature non-interactive |  |  | Nature connectedness was not shown to be a significant covariate in any of the analyses |  |
| Mostajeran 2021 | Four experimental conditions: i) VR urban  ii) slideshow (2D flat screen) urban  iii) VR Forest  iv) slideshow (2D, flat screen) Forest  and iv) one control environment: a silent black virtual room with a white screen in the middle showing a fixation cross. | ) S*ense of presence* for the 360^◦^ was significantly higher than the 2D slideshow conditions; ii) the forest environment induced a higher *sense of presence* compared to the urban environment; iii) for the *sense of being there*, *spatial presence*, *involvement* and *experience* sub-scales, the 360^◦^ image was significantly higher than the 2D slideshow conditions; iv) the forest environment induced a higher sense of *spatial presence* and *involvement* than the urban environment. However, there was no significant effect of immersion level on the outcome, mood |  |  |  |
| Mostajeran 2023 | Two experimental conditions: Computer-generated nature environment vs. abstract objects (cylinders, cubes and cuboids) | Participants in the CGI nature environment had a significantly higher sense of presence compared to those in the CGI abstract objects (cylinders, cubes and cuboids) environment | CGI nature compared to VR abstract objects environment (cylinders, cubes and cuboids) had significantly higher perceived restorativeness |  |  |
| O’Meara 2020 | Experimental (high anxiety) group and a control (low anxiety) group | A link was noted between presence and relaxation for VR, but the link was higher for DVD |  | Nature connectedness was not shown to be a significant covariate in any of the analyses |  |
| Reece 2022 | Two experimental conditions:  1) viewing on flat screen (2D), or  2) viewing on VR,  three environment videos (green, blue historic) | A study included open-ended questions to understand experience of immersion, and reported more positive comments about feeling immersed for the 360^◦^ experimental condition compared to the flat-screen condition |  |  |  |
| Theodorou 2023 | Four experimental conditions:  1) VR urban environment (control),  2) National Park,  3) Lacustrine environment,  4) Artic environment |  | The three 360^◦^ nature conditions (National Park, Lacustrine, Arctic) were significantly more effective compared to the 360^◦^ urban condition in enhancing vitality through restorativeness |  |  |
| Villani 2007 | 4 experimental groups:  1) VR group  2) DVD group  3) Audio group  4) Control group |  |  |  |  |
| Villani 2008 | 4 experimental groups:  1) VR group  2) DVD group  3) Audio group  4) Control group |  | There were no significant differences between participants in the group that had different terrain scenes vs. no change in terrain scene on restorativeness, and there were no significant differences in the group that had natural material pavement vs. those that had artificial materials. There were significant differences in the restorative effects between the different green vision levels, with higher green vision associated with great restorative effect |  |  |
| You 2023 |  |  |  | Participants experienced higher levels of connection with nature in Combination followed by Turbid outdoor green, outdoor green and indoor green compared to the non-biophilic environment |  |
| Zhao 2024 | 3 green components:  1. terrain scene,  2. pavement material,  3. scene green visual rate  1. Terrain scene  In Scenario 1 (experimental group), the terrain was altered, whereas all other factors remained constant. Conversely,  Scenario 2 (control) featured a flat field with no terrain modifications.  2. Paving material  The experiment on terrain material compared Scenes 3 and 4, with the only difference  being the paving material. The other factors in the scenes were kept constant. Scenario  3 (experimental group) used natural materials, such as marble, pebbles, granite, wood, and plain soil, while  Scenario 4 (control) used artificial materials, such as cement, asphalt, glass, steel, and concrete.  3. Green visual rate  The obtained green vision rate was divided into four grades  using the quartile method: lower (<37%), low (37%–42%), high (42%–54%), and higher  green vision | Being in a relaxed state was found to be linked to a sense of presence from VR nature |  |  |  |
